# Current status of ultrasound training in obstetrics and gynecology: a scoping literature review

**DOI:** 10.3389/fmed.2024.1426484

**Published:** 2024-11-12

**Authors:** Julia Matschl, Jorge Jimenez-Cruz, Valentin Sebastian Schäfer, Agnes Wittek, Christoph Berg, Annegret Geipel, Ulrich Gembruch, Brigitte Strizek, Florian Recker

**Affiliations:** ^1^Department of Obstetrics and Prenatal Medicine, University Hospital Bonn, Bonn, Germany; ^2^Clinic of Internal Medicine III, Oncology, Hematology, Rheumatology and Clinical Immunology, University Hospital Bonn, Bonn, Germany; ^3^Division of Prenatal Medicine, Gynecological Ultrasound and Fetal Surgery, Department of Obstetrics and Gynecology, University of Cologne, Cologne, Germany

**Keywords:** obstetrics, gynecology, ultrasound, education, training, ultrasound training

## Abstract

**Introduction:**

As a widely accessible, cost-effective, and safe imaging tool, obstetric and gynecologic (OB/GYN) ultrasound (ULS) plays a vital role in diagnostics and patient care. With its growing relevance, the demand for comprehensive education in this field increases. The objective of this work was to outline the current state of OB/GYN ULS education.

**Methods:**

A scoping literature search was performed until May 2023 using the medical database PubMed according to PRISMA guidelines. Using specific keywords, relevant publications were filtered. Subsequently, abstracts were independently reviewed by two authors and the inclusion of each publication was assessed against pre-defined key search terms. Full-text versions of the included publications were scrutinized and pertinent information was extracted.

**Results:**

In this review, 126 articles from the literature search matched the inclusion criteria and were investigated. Our findings revealed a diverse range of course concepts and programs, a lot of them not meeting the expectations of trainees and international guidelines. OB/GYN ULS training primarily targets residents, yet opportunities for early exposure and continuing education are underexplored. International organizations, such as the International Society of Ultrasound in Obstetrics and Gynecology (ISUOG) and the German Society for Ultrasound in Medicine (DEGUM) have proposed guidelines and curricula for standardized training. However, adoption remains varied. There is an emergent need to innovate teaching methods.

**Conclusion:**

There is consensus that standardizing OB/GYN ULS curricula could enhance training quality and streamline the creation of new programs, ultimately improving patient care. Further research is needed to define the most effective strategies for curriculum development and implementation.

## Introduction

Ultrasonography stands out as a widely accessible, cost-effective, and safe imaging modality, particularly when compared to alternatives like computed tomography and magnetic resonance imaging. These attributes make it the go-to imaging tool in obstetrics and gynecology (OB/GYN). Unlike the latter modalities, however, ultrasound (ULS) imaging is highly operator-dependent. Therefore, adequate technical skills and a thorough understanding of anatomy are essential for performing ULS examinations. In the face of mounting educational requirements and constrained time in residency training programs, dedicated training time for mastering ULS competency has significantly decreased (1). Furthermore, with rapid advancements in ultrasound technology and its expanding applications, continuous education and skill development are imperative for healthcare professionals to remain at the forefront of clinical practice.

Numerous surveys and reviews highlight substantial disparities in curricula across programs within countries and between nations and continents. They emphasize inadequate ULS training and advocate for a standardized curriculum (2–11). In response, various organizations have established guidelines for ultrasound education. The International Society of Ultrasound in Obstetrics and Gynecology (ISUOG) offers both Basic and Advanced Training programs that emphasize practical skills and theoretical knowledge, including patient examinations and written tests such as the “ISUOG Basic Training Test” for assessing theoretical (12, 13). The German Society for Ultrasound in Medicine (DEGUM) has a tiered certification system with Basic (I), Advanced (II), and Expert (III) levels. DEGUM’s certification process is comprehensive, requiring a specific number of supervised scans, a logbook of image evidence, and periodic recertification (12, 14–16). These guidelines, such as those from the Royal College of Obstetricians & Gynaecologists (RCOG), the Society of Obstetricians and Gynaecologists of Canada (SCOG), the Royal Australian and New Zealand College of Obstetricians and Gynaecologists (RANZOG), the Fetal Medicine Foundation (FMF), and several American organizations like the American Institute of Ultrasound in Medicine (AIUM) and the American College of Obstetricians and Gynecologists (ACOG), serve as benchmarks for developing national curricula and evaluating trainee competence, ensuring a high standard of care.

Several systematic reviews have previously examined ultrasound education in OB/GYN, highlighting the importance and challenges of training in this field. For instance, Bidner et al. (17) evaluated antenatal Point-of-Care Ultrasound (PoCUS) training, while Dromey et al. (18), Taksøe-Vester et al. (19), and Woodhead et al. (20) systematically researched the use of ultrasound simulators in obstetric ultrasound education. These reviews, along with others such as those by Lous et al. (21), provide valuable insights into specific aspects of ultrasound education, including simulation training and competency metrics. Despite these contributions, gaps remain in the literature. Previous reviews often concentrate on specific training methods or settings w without offering a comprehensive overview of the broader educational landscape in OB/GYN ultrasound, including all existing teaching methods for example. Additionally, the rapid evolution of ultrasound technology and educational methodologies necessitates an updated synthesis to capture recent advancements and ongoing trends.

The objective of this scoping literature review is to explore the existing body of research focused on ultrasound education in obstetrics and gynecology. By synthesizing available evidence, we aim to identify key themes, common methodologies, and outcomes reported in studies addressing ultrasound education programs, curricula, teaching strategies, and assessment methods. Through a systematic and comprehensive review of the literature, this study seeks to address several important research questions, such as:

- What are the recommended recipients and appropriate stages for OB/GYN ultrasound (ULS) training?
- Who are the qualified instructors for OB/GYN ultrasound (US) training?
- How does the group size impact the effectiveness of OB/GYN ULS training?
- How are ultrasound education programs structured, and what are the core components of these curricula?
- What teaching methods are currently employed in OB/GYN ULS education, and which are considered most effective?
- What are the common challenges faced by trainees during OB/GYN ULS training?
- What is the effectiveness of simulation-based education in OB/GYN ULS training?
- What are the current approaches and applications of Point-of-Care Ultrasound (POCUS) in obstetrics and gynecology?
- What methods are used to assess the skills of trainees in OB/GYN ULS training?

By answering these questions, we aim to provide an evidence-based synthesis of the current state of ultrasound education in obstetrics and gynecology. The findings of this review will contribute to the ongoing efforts to enhance educational practices, develop standardized curricula, and promote best practices in ultrasound training. Ultimately, this research endeavor aims to improve patient care and outcomes through the cultivation of highly skilled and competent healthcare professionals in the field of obstetrics and gynecology.

## Objectives

The purpose of this review was to provide a general overview of the current state of OB/GYN ULS education worldwide, regarding target groups, teaching staff, didactic methods and course formats, also considering previous problems and future opportunities, to support the continuous improvement of the teaching of OB/GYN US.

## Methods

### Search strategy

This scoping literature review was conducted and registered on OSF, with the registration available at https://doi.org/10.17605/OSF.IO/7TEP5. From February 2023 to May 2023, the database PubMed was searched for relevant publications in English or German on the topic of Ob/Gyn ULS education using the keywords (obstetrics), (gynecology), (ultrasound) and (education). Titles and abstracts were screened by two blinded authors for compliance with inclusion criteria. In addition, reference lists of the included articles were investigated for further potential inclusion. All sources were reviewed that pertain to the teaching of OB/GYN ULS to undergraduate students studying for medical degrees and postgraduate medical professionals. Key search terms were used during the literature search, such as “OB/GYN ultrasound education,” “training formats,” “curricula,” and “teaching methods.” The inclusion criteria considered retrospective and prospective studies, surveys, guidelines, recommendations or other publications that concerned the teaching of students, residents or physicians and contained information regarding selected target groups of teaching personnel, course concepts and formats, didactic methods and teaching material used, as well as previous problems and possible opportunities and solutions.

We have chosen to classify these groups into two broad categories: undergraduate students and postgraduate trainees. For the purposes of clarity, undergraduate students will refer to those pursuing medical degrees prior to any formal certification, while postgraduate trainees will encompass those in residency programs or similar post-degree training. Additionally, we will refer to senior medical professionals as staff-grade doctors or consultants, recognizing that not all senior doctors hold the title of professor. By defining and consistently using these terms, we aim to reduce confusion and enhance the precision of the discussion.

No restrictions were made regarding the specialty of the publishing authors. Duplicate articles were excluded. In addition to the literature search in PubMed, the work was supplemented by a targeted search for guidelines such as the International Society of Ultrasound in Obstetrics and Gynecology (ISUOG), the German Society for Ultrasound in Medicine (DEGUM), the Royal College of Obstetricians and Gynaecologists (RCOG), the Society of Obstetricians and Gynaecologists of Canada (SCOG), the Royal Australian and New Zealand College of Obstetricians and Gynaecologists (RANZOG), the Fetal Medicine Foundation (FMF), and several American organizations such as the American Institute of Ultrasound in Medicine (AIUM), the American College of Radiology (ACR), the American College of Obstetricians and Gynecologists (ACOG), the American College of Osteopathic Obstetricians and Gynecologists (ACOOG), the Society for Maternal-Fetal Medicine (SFMF), and the Society of Radiologists in Ultrasound (SRU). The questions that structure this articles as subheadings served as a guide during data extraction.

## Results

**Figure 1:**
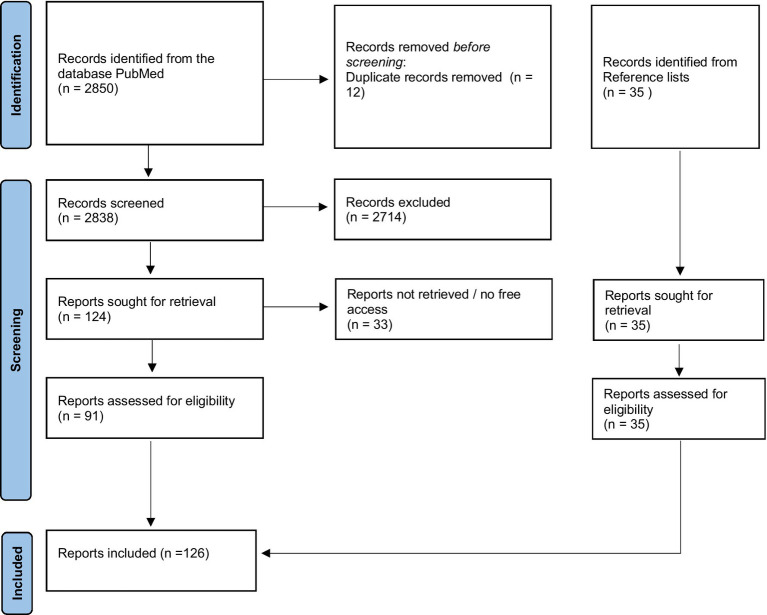
Study selection progress.

Finally, 126 articles, comprising 46 reports, and 80 studies were acceptable for consideration in the literature selection (Figure 1). Supplementary Tables S2, S3, which can be found in the supplementary material, list all reports and studies with respect to their PICOS criteria (Supplementary Table S1).

### Who should receive OB/GYN ULS training and at what stage?

It is widely acknowledged that all OB/GYN trainees should, at a minimum, obtain a foundational understanding of both obstetric and gynecologic ultrasound theory and skills (8). Traditionally, OB/GYN ULS education primarily takes place during residency programs, thus the majority of developed curricula are tailored specifically for postgraduate learners (8, 11, 22–24). Nonetheless, it is highly likely that practicing ULS skills hands-on throughout medical school reinforces acquired knowledge. Real-time visual information enhances comprehension of anatomy, topography, physiology, pathophysiology and the motion of anatomical structures in functional tests like Valsalva. Integrating ULS in preclinical stages may boost knowledge acquisition and long-term benefits (25, 26). The introduction of ultrasound education during undergraduate training requires significant investment in both equipment and faculty training. Furthermore, curriculum space is often limited, necessitating careful planning to ensure that ULS training does not detract from other essential competencies. Evidence from studies on medical education suggests that while early exposure can lead to improved retention of skills, the initial costs (both financial and logistical) may be substantial. Therefore, institutions must weigh these costs against the potential benefits when considering early integration of ultrasound into medical curricula. A cost–benefit analysis, possibly using existing models in medical education, may provide further insight into the feasibility of this approach.

Regarding the long-term effects of preclinical ULS education, a longitudinal ULS curriculum for fourth-year medical students at The Ohio State University was implemented to alleviate instructional demands on physician residency programs and enhance overall physician proficiency. Graduates demonstrated significantly more extensive ULS training than their peers or faculty in residency programs. This suggests that incorporating advanced ULS training in medical school curricula is feasible and supports early access and understanding of the rapidly expanding ultrasound field (27). Similarly, The University of South Carolina successfully introduced an integrated ultrasound curriculum (iUSC) across all 4 years of medical school, greatly enriching their students’ medical education as affirmed by positive feedback (28). The short-term effects of introducing basic ULS screening in undergraduate medical school have also been demonstrated in a study by Hamza et al. (29) through improved student knowledge and image recognition. Students expressed high satisfaction and a desire for more hands-on sonographic training in OB/GYN and other medical fields. A survey by Kessler and Bhandarkar (30) emphasized the demand for additional ULS training among medical students and residents, and numerous sources advocate for implementing such training as early as possible in medical school (31, 32). Obstetric ULS training is also feasible in an interprofessional setting, including participants from various healthcare professions such as registered and advanced practice nurses, midwives, physicians, as well as nursing and medical students. This approach fosters collaboration, diminishes hierarchy, and particularly benefits student participants with little to no prior OB ULS exposure, supporting the idea of incorporating ULS curricula early in medical education (33).

### Who should teach OB/GYN US?

Learning ULS is most effective through hands-on practice in small supervised groups (25, 26). However, this method can be labor-intensive, time-consuming, and costly, especially when overseen by senior residents or professors (postgraduate trainee). To overcome this issue, teaching formats with a multiplier effect can be utilized, such as the “Teach-the-Teacher” method where senior staff train peer tutors to teach practical skills to their peers (25). Notably, the efficacy of „peer teaching “is not inferior to faculty-led instruction (34, 35). A study by Dornhofer et al. (36) underscores the efficacy of peer teaching in a point-of-care-ultrasound (POCUS) curriculum, including OB/GYN US. Medical students effectively teach peers, physicians, nurses, and midwives, leading to significant knowledge absorption and practical ULS skills. This highlights the value of peer teaching and challenges the belief that only academic experts can provide ULS instruction. Student-led teaching promotes learning and creates a positive working atmosphere by establishing social and intellectual connections (37) and enhance the peer tutors’ own knowledge and skills (35). In conclusion, peer teaching is a well-established, proven, and widely accepted approach that facilitates undergraduate ULS training for a broad range of students, ultimately making it more accessible and efficient (38).

### Does group size matter?

As the number of medical students worldwide continues to grow, resulting in larger class sizes, cost-effective and resource-efficient approaches like dyad training (i.e., learning in pairs) are becoming increasingly popular, particularly for the acquisition of clinical skills. However, concerns about potential compromises to the quality of education are prevalent. Recent research indicates that compared to individual learning, dyadic learning in simulated settings enhances skill retention and boosts confidence for future patient interactions (39). Furthermore, training efficiency could be doubled without jeopardizing the skill transfer from simulation-based training to complex clinical interactions with patients (40).

Noerholk et al.’s study (41), which compared individual training with dyad, triad and tetrad training, revealed that cooperative skill learning in groups of up to four people did not negatively affect skill transfer, despite the reduced hands-on time. This outcome might be due to a compensatory increase in constructive and interactive learning activities, offsetting the effect of reduced hands-on experience.

Complementing this, a 2022 qualitative study by Windrim and Higgings (42) explored trainees’ experiences of learning transvaginal ULS via simulation, juxtaposing dyadic and individual training methods. Dyad training received significant approval based on participant interviews. Trainees appreciated having a partner for problem-solving, mutual encouragement and learning from mistakes. However, individual learners preferred self-paced learning but faced difficulties in error detection. These findings align with previous qualitative research (43) and quantitative studies (39, 41), endorsing dyadic training as an effective pedagogical approach, with individual training best suited for learners needing a more tailored learning speed.

Several theories underscore the potency of dyad training in the realms of motor skill acquisition, neuroscience, and psychology. The peer-assisted learning theory, for instance, explains how collaborative learning boosts effectiveness through enhanced confidence, shared memory, and cognitive partnership (44). Existing literature and empirical studies on motor skill learning posit that the benefits of dyad practice primarily stem from observation (45–50), and neuroscience research indicates that the brain uses the same neural pathways for both observing and actively executing actions, primarily involving “mirror neurons” (51, 52). Moreover, the cognitive load theory suggests that dyad practice can reduce the risk of cognitive overload by sharing information processing and collective memory, thereby enhancing learning efficiency (53). Collaboration is valuable for learners in the early stages of skill acquisition when cognitive load is high. However, as proficiency improves and cognitive load decreases, the benefits of collaboration diminish. More hands-on practice is necessary to develop skill automaticity. At this stage, individual training outweighs the advantages of shared cognition and cognitive co-construction. This shift from collaborative to individual learning is supported by evidence from various fields, including medicine (45, 46, 48, 49).

### What teaching methods should be incorporated into an OB/GYN ULS curriculum?

The traditional method of clinical training involves workplace-based learning through apprenticeships, where trainees acquire knowledge through observation, supervision, and independent learning. However, implementing this approach is challenging due to its opportunistic nature and reliance on self-direction. This has raised concerns about its effectiveness for basic clinical training (54). Tolsgaard et al. (55) found significant gaps between expected performance levels and perceived abilities, suggesting that clinical apprenticeship training may be insufficient without dedicated time for foundational training. Trainees did not perceive frequent supervision requests as detrimental to their credibility and in fact indicated a desire for more supervised practice.

Other traditional teaching methods include didactic presentations or lectures for large audiences. These are suitable for teaching fundamental ULS principles, including physics, knobology (i.e., machine operations and controls), standard ULS terminology, safety concerns (e.g., the “As Low As Reasonably Achievable” principle), and examination techniques, along with relevant anatomy, physiology, and important pathologies. To stimulate active learning, the lecture format can be enhanced by incorporating an audience response system. However, as revealed by a study conducted by Tregonning and et al. (56), this approach showed only temporary benefits compared to conventional didactic lectures, without demonstrating long-term effects. Another interactive teaching method is case-based learning. This pedagogical approach encourages learners to engage actively in real-time problem-solving exercises centered on specific clinical cases. It has been found to be especially advantageous for learning “Very Important Pathologies” (VIPs) (25).

But the key to gaining proficiency in ULS lies in observation and practical, hands-on courses that enhance student motivation through simulated or real patient scanning, fostering the development of psychomotor abilities (1). These skills can be defined as the “unique mental and motor activities required to execute a manual task safely and efficiently for each clinical situation” (57). Visuospatial skills, the mental component, involve creating mental 3D-images of anatomy or anomaly from 2D representations and guiding the transducer to a target location. Motor activities pertain to the coordination of movement in tandem with visual input, often referred to as hand-eye coordination or visuomotor skills. Both skill sets depend on learners having a visual exemplar of standard performance for reference and assessment of anatomic structures (57). Consequently, integrating clinical data and represented anatomy or physiology with real-time ULS images constitutes one of the most captivating and well-received training methods (25).

Observing ULS examinations, a key component of traditional apprenticeship, has limited effectiveness in helping learners master the subtle motor movements required for task execution. The best pedagogical strategies for teaching complex psychomotor skills are still uncertain, but it is clear that extensive hands-on training and regular supervision are crucial for acquiring them (51–54). Conventional apprenticeship, even with additional lectures, often leads to insufficient practical training and inadequate image acquisition skills. Medical schools should consider revising their curricula and adopting innovative teaching methods to address this issue (25, 58).

Innovative learning methods include peer teaching, concise information delivery, hands-on workshops, supplementary resources, and educational media. ULS skills labs and simulators are crucial in this innovation, offering a safe environment for learners to practice without risking patient harm. Simulators replicate diverse clinical scenarios, providing experience in managing complex situations. When teaching complex skills, it is recommended to break them down into smaller components to avoid cognitive overload, considering limited working memory capacity (58, 59). Peyton’s four-step approach, which encompasses demonstration, deconstruction, comprehension, and performance, is a modern alternative to the traditional “see one, do one” method and has been validated as an effective method for procedural skill acquisition (60).

To enhance student preparation for practical aspects of the course, a range of accessible study materials such as textbooks, e-books, video lectures, apps, and interactive e-learning tools are recommended. These resources can be used alongside practical coursework to cater to different learning styles (25). Multimodal training approaches that combine digital resources with hands-on courses have demonstrated effectiveness in various settings (61, 62). For example, McCurdy’s (63) test showed initial benefits, but these gains were not sustained in the follow-up assessment after 6–10 months. To enhance self-directed learning, it is suggested to introduce a web-based learning portfolio, allowing trainees to document patient interactions and reflect on significant learning incidents (64). Additionally, computer-based learning methods outperformed traditional paper-based ones in post-tutorial exams (65). E-learning modules, which allow students to personalize their study pace, duration, and setting, are generally well-received (33). These modules are invaluable in regions with limited educational resources, showcasing their global relevance. Asynchronous e-learning is beneficial to support student preparation, reflection, and knowledge reinforcement. Additionally, integrating pathway exams into e-learning frameworks allows students to independently assess their knowledge (31).

**Figure 2:**
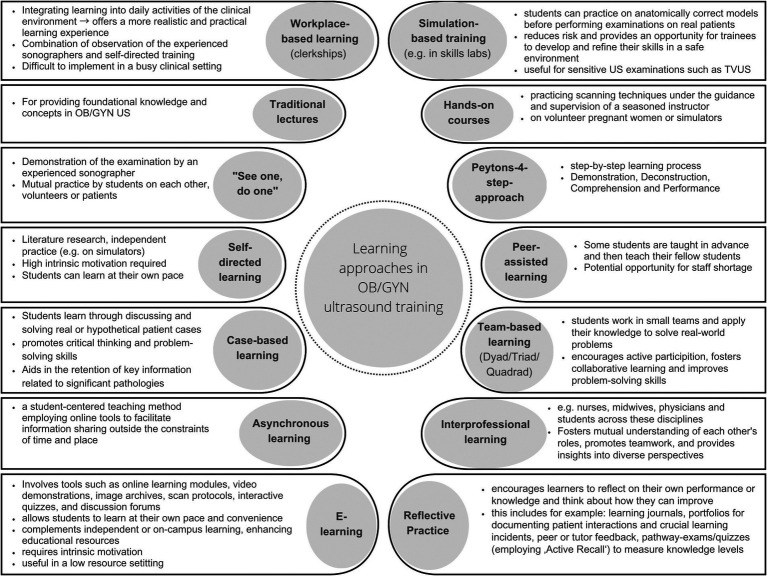
Summary of different learning approaches in OB/GYN ULS training and their characteristics.

Given that ULS expertise relies heavily on pattern recognition, resources such as image archives are essential for skill development (25). As early as 1995, Lee (66) explored the potential advantages of employing interactive multimedia tutorials, a dedicated ultrasound library, and volume visualization models for prenatal ultrasound training. More recently, tools like the “Pocket Brain,” an online multimedia atlas focused on fetal brain anatomy and pathology, offer novel platforms for instruction and learning (67). Similarly, the Visual Encyclopedia VISUOG integrates various teaching modalities through an anatomical approach^1^ (68). Such innovative tools underscore the growing significance of multimedia and interactive tools in medical education. Figure 2 presents a summary of various learning approaches in OB/GYN ULS training, including their key characteristics.

#### Enhancing and sustaining ULS quality

To enhance or sustain acquired ultrasound abilities, feedback on examination quality through accreditation training or clinical audits, along with targeted actions like additional training sessions when necessary, has proven beneficial (69–71). Ultrasound and psychomotor skills can be learned to a certain proficiency level. However, without reinforcement or regular use, these skills may decline below competency thresholds. Feedback is crucial to help novices develop scanning skills and assist experienced sonographers in refreshing or regaining their abilities. Quality assurance, including clinical audits, is essential as it is closely linked to patient safety and accreditation. The Northwell Health Ultrasound Task Force recognizes the importance of prioritizing quality assurance in maintaining patient safety and quality (32). Automating the process could yield further advantages, especially in terms of time savings (70). The AIUM offers accreditation for OB/GYN ultrasound through a peer-review process, ensuring adherence to nationally recognized standards outlined in joint guidelines with the ACR. The Ultrasound Practice Accreditation Council (UPAC) evaluates practices’ policies and procedures to safeguard patients and personnel. It promotes self-reflection, internal quality assurance protocols, and assessment of members’ education and training (32). The German Ultrasound Society (Deutsche Gesellschaft für Ultraschall in der Medizin, DEGUM) has instituted a three-tier system to ensure consistent quality and expertise among practitioners. Unlike the voluntary accreditation programs of AIUM or UPAC, DEGUM requires practitioners to recertify every 6 years to uphold high standards of care (degum.de) (72).

### What challenges do trainees encounter during OB/GYN ULS training?

The quality of ULS examinations is significantly operator-dependent due to the extensive learning curve associated with mastering complex ULS procedures (8). Trainees typically need 24 months of clinical experience or 12–24 days in a specialized ultrasound unit to confidently perform ULS examinations, as demonstrated by Tolsgaard et al. (55). Ultrasound confidence is influenced by three factors: technical proficiency, image interpretation, and integration of the scan into patient care, which encompasses a blend of motor, visual, and cognitive skills. Technical skills, in particular, are often ranked as subpar (55). Based on several findings (55, 73, 74), one could postulate that while basic training might enhance technical performance, clinical training alone may be insufficient for achieving mastery learning. Simulation-Based Medical Education (SBME) can effectively teach fundamental ULS techniques and serve as a valuable supplement to clinical training, as further detailed in the following paragraph (75).

### Is simulation-based education effective for OB/GYN ULS training?

In general, simulation can be defined as a “technique to replace or amplify real experiences with guided experiences that evoke or replicate substantial aspects of the real world in a fully interactive manner” (76). ULS simulators include a human simulation model and a mock probe connected to a computer monitor. The monitor displays ULS images based on the probe’s position and movement. These simulators are valuable for training in transabdominal and transvaginal OB/GYN US, as well as for evaluating trainee competence (77).

#### Immediate and long-term learning effects

Research on SBME has primarily focused on its immediate outcomes and advantages, such as enhancements in knowledge, performance, image quality, and diagnostic accuracy (78–89). Some studies have explored skill transfer, the application of previously acquired knowledge or abilities to new challenges or contexts. However, these investigations typically assessed short-term effects rather than long-term impacts (40, 90). Only a limited number of studies have examined the sustained skill transfer in SBME.

A significant study in this context is a randomized controlled trial by Tolsgaard et al. (91) conducted in 2015.

This study examined the impact of initial simulation-based transvaginal ultrasound (TVUS) combined with clinical training compared to clinical training alone on clinical performance after 2 months. The findings demonstrated that integrating simulation-based training with clinical training resulted in significant and lasting improvements in clinical performance over 3 months. However, while this suggests that combining the two approaches may yield better outcomes than clinical training alone, it does not establish simulation-based training as inherently superior to clinical training (75).

In contrast, Le Lous et al. (92) explored the impact of SBME on the quality of ULS images obtained by general practice residents 2 months post-training, compared to clinical training alone. Their study demonstrated significant improvements in the quality of pelvic ULS images among residents who underwent simulation-based training before their two-month gynecological experience. However, a prospective randomized study by Grandjean et al. (93) did not find long-term benefits of SBME. The study, which compared a six-month course integrating a simulation-based workshop on fetal biometry in ULS to the same course without the workshop, found no significant differences in skill enhancement between the groups.

Critiques of the studies by Tolsgaard et al. (91) and Le Lous et al. (92), which investigated the “sustained effects” of SBME, suggest that it may be premature to discuss about sustained effects just 2 months post-simulation. A more comprehensive comparison would juxtapose exclusive SBME against exclusive clinical training. Adding weight to this critique, a study by Moak et al. (87) revealed lower performance scores among students who practiced ULS skills on a pelvic mannequin compared to those who practiced on live models, when assessed on standardized patients. This naturally raises a pertinent question: can or should exclusive simulation-based ultrasound training replace clinical practice? The prevailing consensus is no. SBME is typically viewed as an adjunct tool for enhancing OB/GYN ULS education, establishing a standardized quality of training while significantly improving the examiner’s skills (88).

Interestingly, a study by Katz et al. (94) evaluated a computerized interactive simulator combined with instructor supervision and web-based immediate feedback to measure its effectiveness. The results indicated that having an instructor involved in the simulation-based training led to improved learning outcomes. Additionally, the study found that different training content and trainee populations did not affect the overall learning gains. These findings support the notion of integrating multiple teaching approaches, particularly emphasizing the inclusion of an instructor who provides feedback.

#### Trainees’ perception

Trainees often struggle to attain minimal ultrasound competencies with clinical training alone, which is why they express a positive perception toward incorporating ultrasound simulation into curricula (89). In previous exploratory studies, they have communicated their eagerness for ULS simulation as a teaching tool, believing it will shorten their learning curve and enhance their clinical skills and knowledge (42, 95, 96).

#### The aspect of time

A noteworthy finding from the study by Grandjean et al. (93) is the importance of the timing at which a simulation-based workshop is incorporated. Their qualitative analysis confirmed that embedding SBME during the early phase of a practical course enhances the minimal proficiency within a group. This is in contrast to incorporating SBME before or in the late stage. Traditional ULS training during clerkships or internships can be time-consuming and challenging to accommodate in a busy clinical environment. On the other hand, simulation-based ULS training enables novices to learn skills more efficiently within a shorter timeframe. The rapid improvement of ULS skills among beginners or obstetricians with minimal experience suggests that short phase virtual reality (VR) simulation training could serve as an effective warm-up exercise before clinical sessions, minimizing disruption to clinical services (81).

Etienne et al.’s study (84), which examined the impact of initial TVUS training, further highlights the benefits of incorporating SBME early in the training process. Regarding the concept of brief VR simulations, one may wonder how long it takes for a novice to achieve expert level. In 2014, Madsen and et al. conducted a study (85) that explored the learning curve associated with using a VR simulator for TVUS. The study concluded that beginners' performance undeniably improved with practice, and their learning curves plateaued at the level of expert performance after approximately 3–4 h of simulator training. Similar to a study by Dyre et al. (97), which found less than 5 h until mastery learning levels were achieved.

In conclusion, short-phase VR simulations introduced early in the training process can lead to significant performance improvements. But when is the best time to incorporate simulation-based ULS training into medical education? Cook et al. (83) demonstrated that even third-year undergraduate medical students can benefit from early exposure to simulation-based ULS training. Similarly, Andreasen et al. (80) found substantial value for seasoned practitioners. Simulation-based ULS training enhanced the accuracy and image quality in fetal weight estimation for women at term, regardless of the obstetrician’s level of clinical experience.

#### Safe teaching environment and theoretical foundations

Simulation-based training allows repeated practice within a safe environment, permitting errors until proficiency is achieved (87). This method emulates a clinical environment, granting the instructor direct control over the trainee—an aspect often unattainable in hectic clinical settings (76, 98). Combining repeated practice with expert supervision promotes *deliberate practice*, which is regarded as a critical determinant for acquiring expertise across various domains (99). Consequently, SBME offers an optimal environment for *deliberate practice*, serving as the foundation for effective learning within SBME (100). Through SBME, errors can be committed without putting patient well-being at risk, helping to avoid negative outcomes such as deaths, misdiagnoses, complaints, or claims (101). The negative emotions arising from these mistakes, crucial to the medical learning process, are experienced more constructively within a simulated environment than in real life (102). Additionally, numerous studies report substantial increases in the trainees’ confidence and comfort following SBME (78, 82, 83).

#### Quality and efficiency of care

The influence of SBME on quality and efficiency of patient care was probed via a multicenter randomized trial (103). The key question was, “How does initial simulation-based TVUS training impact quality and efficiency of care during the first 6 months of practice compared to traditional clinical training alone?” While SBME improves knowledge, skills, and behavior significantly, its effect on patient outcomes is only moderate, consistent with Cook et al.’s meta-analysis (104). Still, these “moderate effects” included a better quality of care, reduced patient discomfort, increased patient-perceived safety, as well as decreased necessity for repeated examinations and trainee supervision (103). Further, the potential to replace live ULS models with simulators was explored by Bentley et al. (105) in the context of trauma scans. They found that simulated training was as effective as live model training, suggesting that live models could be substituted in certain scenarios, aligning to similar results in Rosen et al.’s study (89). On another note, Graber et al. (106) found that patients were more likely to consent to procedures after students underwent simulation training. In contrast, trainees in the early stages of conventional training may frequently encounter patient refusal for examination. This is particularly relevant for TVUS, a sensitive procedure with limited training opportunities due to its intimate nature. Since practicing such examinations as TVUS on real patients is neither feasible nor ethical, and it is an essential examination method for early pregnancy assessments and other OB/GYN concerns, simulation training can serve as a vital solution to bridge this learning gap (107).

#### Monetary concerns

One major hurdle in integrating ULS simulations into OB/GYN education is the potential high costs associated with procuring equipment and supervision. Though generally less costly than live models, simulation-based training still tends to be more expensive than traditional apprenticeship (89). Tolsgaard et al. (108) approached this issue by developing a cost-effectiveness model for health education, with simulation-based ULS training as a case study. The study found that even significant educational results do not guarantee cost-effectiveness, and adopting new training methods depends on a balance of costs, effectiveness, and willingness-to-pay. Efforts to develop more cost-effective ULS simulators have also been made. For instance, the fetal pig simulator proposed by Nitsche and Brost (109) uses fetal pigs of varying sizes sealed in clear, formalin-filled plastic bags. Trainees can obtain clear images by placing the ULS probe with an adequate amount of gel directly onto the bag. Despite its cost-effectiveness, this model has limitations due to the obvious anatomical differences from a human fetus. Akoma et al. (79) conducted a randomized controlled trial assessing the impact of this inexpensive, anatomy-based fetal pig simulator on OB ULS training. No differences were found in post-course biometric scans between hands-on scanning with pregnant women and hands-on scanning with the fetal pig simulation. However, significant improvements were observed in scan completion time and the number of technically adequate images obtained with simulation training. The issue of cost-effectiveness remains challenging and monetary concerns persist despite these efforts.

### Point-of-care ultrasound in OB/GYN

POCUS has revolutionized the medical field, particularly in OB/GYN, providing real-time scans at the bedside for diagnostic and therapeutic purposes. These scans offer rapid, safe, and precise methods for assessing fetal development, diagnosing gynecological issues, guiding invasive procedures, aiding physical exams, and providing immediate feedback in emergency situations. Although its use spans across clinical settings from prenatal care to labor and delivery, its formal teaching in OB/GYN residency lags behind other medical specialties (110, 111). The call for incorporating POCUS into medical student curricula and residency programs for OB/GYN is underscored by research. For instance, a study by Vyas et al. (112) demonstrated the successful teaching of a rural ultrasound triage exam, the “ROUTE,” to first-year medical students. This method can be used to screen high-risk conditions in pregnant women, which allows women to obtain further care as needed, particularly in resource-limited settings. Further evidence comes from Dornhofer et al.’s study (36), indicating that medical students can effectively teach POCUS to healthcare professionals in rural settings. The study showed significant uptake of knowledge and practical ultrasound skills post an intensive 4-week course, reinforcing the value of POCUS education. In line with this, the EFSUMB position paper (113) asserts that the affordability and convenience of handheld ultrasound devices make them ideal training tools. With the growing demand for ULS training, it becomes even more crucial to provide this education to medical students. This training not only enhances anatomical understanding but also promotes rapid diagnosis and decision-making during clinical training.

### Skills assessment

Blending simulation-based teaching, live instruction, and diverse teaching methods has enhanced the learning experience in ULS education for countless students. However, skill and proficiency assessment remains a complex component of education. Traditional quantity-based indicators, such as the number of ULS scans completed by a student, may not accurately represent their competence as compared to performance-based evaluations, which are often conducted through direct observation in clinical scenarios or assessments in simulated environments (114).

VR simulators provide a realistic scanning experience, standardizing the teaching, training, and evaluation of ULS skills (78, 115). This uniformity is essential due to the considerable demand for consistent assessment methods and criteria across examiners, institutions, and countries (74). While the time required for trainees to achieve expertise in ULS performance varies, simulator-based solutions present promising opportunities (85, 97). Built-in simulator metrics facilitate automatic evaluation of trainee performance, quantifying progress, and providing targeted feedback for improvement (116, 117). This efficient method allows for the evaluation of numerous students without requiring individual evaluations by sonographers (118). However, these metrics, often referred to as the “Achilles Heel” (77) of simulators, require careful scrutiny, as studies indicate that only about a third can effectively differentiate between novices and experts (73, 117, 119). Despite this, strong evidence exists supporting the validity and reliability of simulation-based assessments of competence for both transabdominal (73, 97, 117, 119, 120) and transvaginal ultrasound (73, 85, 121). For instance, Chalouhi et al. (119) demonstrated that an OB ULS simulator is as effective as volunteer-based examination for evaluating practical skills, while Madsen et al. (85) found similar results for a transvaginal ultrasound simulator.

A more conventional method to gauge students’ learning progress is administering pre- and post-course tests or surveys (29, 35, 38, 40, 41, 62, 63, 65, 79, 80, 82–84, 88–90, 93, 105, 122–124). Initial knowledge is usually measured through multiple-choice or written tests, and results are compared with scores from similar tests at the end of the course (29, 38, 62, 63, 65, 83, 105, 122, 124, 125). While these tests are effective for assessing theoretical knowledge, they fall short in evaluating hands-on skills required in ULS operation, such as image interpretation, scanning technique, and patient interaction.

Some studies have employed the “Image Rating” technique, where experts evaluate ULS images from students based on set criteria, often comparing pre- and post-training scans (26, 34, 69, 80, 84, 89, 92, 119). Although this technique is beneficial for assessing image quality, it should be complemented with practical exams like Objective Structured Clinical Examinations (OSCEs) (28, 35, 105, 124) or Direct Observation of Procedural Skills (DOPS) (62, 71, 93). OSCEs test students’ clinical skills through specific tasks in simulated scenarios, whereas DOPS involve real-time observation and feedback as a student performs a clinical procedure on a real patient (118).

For direct trainee observation, standardized checklists or generic rating scales have been devised to offer standardized, valid competence measures that can be compared across institutions and countries. The most prevalent scale is the Objective Structured Assessment of Ultrasound Skills (OSAUS) (40, 41, 71, 80, 82, 90, 91, 93, 103), a comprehensive and extensively validated tool used to assess general ULS skills in various clinical settings and disciplines, including both transvaginal and transabdominal ULS capabilities (73, 74). Despite criticisms of its general approach rather than procedure-specific focus, the OSAUS is time-efficient and eliminates the need for creating new protocols for different specializations.

In addition to simulator metrics and traditional tests, self-assessments through questionnaires or surveys are frequently used to gauge students’ perception of their own skills, knowledge, and performance (43, 64, 123, 126). Many studies also incorporate a survey to evaluate participants’ course experience and satisfaction (29, 35, 38, 43, 79, 80, 82–84, 87, 88, 96, 125, 127), or to collect subjective data about their comfort or confidence levels throughout the course. Self-assessments, although subjective, are cost-effective and can motivate students to improve by identifying performance gaps. Integrating multiple assessment methods balances the limitations of each format, addresses learning objectives comprehensively, and identifies performance inadequacies. This holistic approach to student evaluation encompasses theoretical understanding and practical skills in real-life scenarios.

## Discussion

Ultrasound education constitutes a pivotal aspect of OB/GYN training, integral to diagnostics and patient care. Therefore, it is essential to continuously refine and expand ULS curricula, incorporating the guidelines established by organizations such as ISUOG and DEGUM. These guidelines provide a framework for structured training programs and certification criteria, ensuring a high standard of competence among practitioners.

Incorporating diverse teaching personnel, including peer teaching from student tutors, presents an efficient, resource-saving approach to ultrasound education.

Differentiating ultrasound training based on the educational level of learners is critical for optimizing learning outcomes in gynecology and obstetrics. Medical students, who are building foundational clinical knowledge, would benefit from a broader curriculum that includes general ultrasound principles and applications across various medical fields. In contrast, residents, who are specializing in gynecology and obstetrics, require more focused, advanced training tailored to the specific complexities of their specialty. By adapting the content and depth of training to match the learner’s stage of education, training programs can more effectively develop both basic and specialized competencies. This tiered approach could enhance the overall efficiency and effectiveness of ultrasound education, ensuring that each group is equipped with the appropriate skills for their level of expertise.

Before transitioning to real-world ULS examinations, trainees may benefit from supervised or self-directed training on ULS simulators. These tools provide hands-on experience in a controlled setting, mitigating the risk of patient discomfort. This preemptive mastery of skills equips practitioners for safer and more effective patient interactions. Notably, studies emphasize the value of simulation-based training for both novice and seasoned operators (80).

Moreover, given the complexity of ULS techniques, trainees can experience cognitive overload. Traditional apprenticeships often struggle to provide the necessary extensive hands-on training, frequent feedback, and effective teaching approaches like Peyton’s 4-step method to divide these complex skills into manageable chunks. Consequently, we propose integrating supplementary hands-on ULS courses into the curriculum, employing volunteers or simulators. This approach is particularly useful for intimate examinations, such as transvaginal ultrasounds, and obviates the need for pregnant volunteers for OB scans.

In line with our review’s findings, we advocate for innovative and flexible strategies in educational design. Traditional lecture-based courses should be enhanced or replaced with active learning strategies, such as case-based teaching, simulation training, and competency-based education. E-learning and image archives, particularly those that include pathological findings, are valuable tools for complementing hands-on training. These resources not only facilitate skill acquisition but also enrich understanding of a wide spectrum of OB/GYN conditions. A multimodal training concept addresses various learning styles and emphasizes hands-on experience – a fundamental element in attaining ultrasound proficiency.

Integrating ULS courses early into medical curricula, with strong focus on practical training sessions, has shown to be more effective than solely relying on traditional apprenticeships or residency programs. This strategy reduces the educational pressure on physician residency programs and potentially heightens overall physician competency. To further optimize the effectiveness of ULS courses, it is recommended to begin with a collaborative learning approach during the initial stages of skill acquisition. As trainees progress toward mastery, a transition toward individual training becomes increasingly beneficial.

Assessing trainee competence necessitates a blend of evaluation methods, going beyond simply counting a set number of scans. Comprehensive skill assessments should incorporate theoretical knowledge examination via multiple-choice questions or written exams, practical skills testing through Objective Structured Clinical Examinations (OSCEs), and use of the Objective Structured Assessment of Ultrasound Skills (OSAUS). Furthermore, Image Rating-based image quality assessments can offer valuable insights into trainees’ capabilities.

It is important to strive for consistency in educational approaches across universities and countries to ensure a baseline level of competency. However, it is also crucial to acknowledge the diversity of learners, contexts, and resources, which may necessitate adaptations to curricular content and delivery methods. Instead of advocating for a completely uniform approach, we propose developing flexible guidelines that can be tailored to accommodate varying educational settings and learner needs. Frequent updates to curricula should be informed by evolving scientific knowledge, clinical guidelines, and feedback from educators and learners. While standardized curricula have traditionally targeted post-graduate OB/GYN training (8, 11, 22–24), emerging evidence supports the early integration of ultrasound education at the undergraduate level to reinforce foundational concepts in anatomy, physiology, and pathology (25, 27, 28).

## Conclusion

Ultrasound education in obstetrics and gynecology must evolve into a structured, comprehensive system that integrates early exposure, hands-on training, and consistent competency assessment. We advocate for incorporating ultrasound training into both undergraduate and postgraduate curricula, starting with foundational concepts early in medical education. This early integration improves anatomical understanding and skill retention, while postgraduate training should include theoretical instruction, simulation-based learning, supervised hands-on practice, and certification. To enhance learning, innovative methods such as peer teaching, simulation, and e-learning should complement traditional didactic lectures. Ultrasound simulators, in particular, allow for risk-free practice, enabling trainees to master essential psychomotor skills. Continuous feedback and clinical audits will ensure sustained competency and quality. Standardizing ultrasound curricula across institutions, with flexible frameworks to accommodate different settings, is crucial for ensuring baseline proficiency and improving patient outcomes. Further research should focus on optimizing teaching methods and assessment practices to meet the evolving demands of ultrasound in clinical practice.

The ultimate aim is to bridge the gap between advancing ultrasound technology and clinical expertise, ensuring practitioners are fully equipped to improve maternal and fetal health through effective diagnostic use.

## Footnotes

^1^ isuog.org
