# Supplementary material for: Current status of ultrasound training in obstetrics and gynecology: a scoping literature review
Source: Front Med (Lausanne). 2024 Nov 12;11:1426484. doi: 10.3389/fmed.2024.1426484 (PMC11588472; doi:10.3389/fmed.2024.1426484)
Supplement: Supplementary file 1 [file Table_1.docx]

**Supplementary Table 1** Included studies (retrospective, prospective, prospective observational, prospective interventional, prospective randomized, prospective cohort, prospective mixed-method, cross-sectional, associational, and experimental studies) and their characteristics

| Author(s) | Title | Medical specialty | Participants | Intervention | Comparison | Outcomes | Study design | Year of publication |
| --- | --- | --- | --- | --- | --- | --- | --- | --- |
| Ahmadzia et al.^47^ | Teaching obstetric ultrasound at Mulago Hospital - Kampala, Uganda | OB/GYN | Medical students, junior and senior house officers  (n = 40) | 1. Pre-course survey 2. Didactic and practical hands-on teaching session 3. Post-course survey | - | Effective method to improve knowledge and skills for medical students and house officers at Mulago Hospital in the area of basic OB US | Prospective study | 2018 |
| Akoma et al.^48^ | Impact of an Inexpensive Anatomy -Based Fetal Pig Simulator on Obstetric Ultrasound Training | OB/GYN | Physicians and non-physician healthcare professionals  (n = 24) | US course   1. Fetal pig simulation (n = 12) | 1. Hands-on scanning with pregnant women   (n = 12) | The fetal pig ULS task trainer resulted in improvements in the course participants’ scanning efficiency even after very limited exposure. Incorporating the task trainer earlier and more broadly into OB ULS training may benefit trainees. | Prospective randomized study | 2015 |
| Alrahmani et al.^49^ | The Current State of Ultrasound Training in Obstetrics and Gynecology Residency Programs | OB/GYN | Residency program directors and residents  (n = 530) | Survey | - | Lack of clearly defined milestones in ULS training in OB/GYN residency,  substantial heterogeneity in curricula between programs, highlighting a need for a standardized ULS curriculum | Cross-sectional study | 2018 |
| Amesse et al.^50^ | Evaluation of Computer-aided Strategies for Teaching Medical Students Prenatal Ultrasound Diagnostic Skills | OB/GYN | Medical students  (n = 36) | Instruction in prenatal ULS diagnostics using interactive computer-based program learning (CBL)  (n = 18) | Instruction using equivalent material by the traditional paper-based method (PBM)  (n = 18) | Students that received CBL had significantly higher post-tutorial exam scores than those in the PBM group, indicating that CBL is an effective instruction strategy in this setting. | Prospective randomized study | 2008 |
| Andreasen et al.^51^ | Multicenter randomized trial exploring effects of simulation-based ultrasound training on obstetricians’ diagnostic accuracy: value for experienced operators | OB/GYN | OBs with different levels of clinical experience  (n = 74) | Simulation-based ULS training focusing on fetal weight scans  (n = 39) | No intervention  (n = 35) | Simulation-based ULS training improved accuracy and image quality when performing fetal weight estimation in women at term, independent of obstetricians’ clinical experience. | Prospective randomized study | 2020 |
| Arthur et al.^52^ | Dyadic Versus Individual Training Protocols: Loss and Reacquisition of a Complex Skill | Psychology | right-handed male volunteers  (n = 177) | 1. AIM (Active interlocked modeling)-dyad protocol (n = 40) | 1. Standard individual protocol (n = 49) | Despite half as much hands-on practice, dyadic trainees did not differ from individuals on tests of skill acquisition, loss after an 8-week nonpractice interval, and reacquisition of a complex skill. | Prospective randomized study | 1997 |
| Bahner and Royall^53^ | Advanced Ultrasound Training for Fourth-Year Medical Students: A Novel Training Program at The Ohio State University College of Medicine | US education | Medical students  (n = 150) | Multimodal longitudinal advanced ultrasound curriculum |  | feasible model for meeting training guidelines without increasing the educational requirements for residency programs | Prospective study | 2013 |
| Bentley et al.^54^ | Are Live Ultrasound Models Replaceable? Traditional versus Simulated Education Module for FAST Exam | Emergency medicine | Medical students  (n = 93) | 1. Training on an ULS simulator (n = 39) | 1. Traditional training on a human model (n = 54) | no difference between groups in knowledge based ULS test scores, survey of comfort levels with US, and students’ abilities to perform and interpret FAST on human models. | Prospective randomized study | 2015 |
| Burden et al.^55^ | Validation of Virtual Reality Simulation for Obstetric Ultrasonography A Prospective Cross-sectional Study | OB/GYN | Trainees and experts  (n = 26) | VR simulation crown-rump length (CRL) ULS scan and growth ULS scan   1. Trainees (n = 18) | 1. Certified experts (n = 8) | Clinicians with differing ULS expertise showed differing skill with the UltraSim VR simulator, demonstrating construct validity for skills needed in simulation. | Cross-sectional study | 2012 |
| Burden et al.^56^ | Usability of virtual-reality simulation training in obstetric ultrasonography: a prospective cohort study | OB/GYN | 18 trainees, 8 certified OBs  (n = 26) | VR simulation training   1. Trainee group (n = 18) | 1. Certified experts (n = 8) | Fast improvement of ULS skills with short phase VR simulation training for OBs with minimal experience 🡪 suggests that VR simulation might be useful as a warmup exercise before clinical training | Prospective cohort study | 2013 |
| Byford et al.^57^ | Implementing the transvaginal ultrasound simulation training (TRUSST) programme for obstetric registrars | OB/GYN | OB registrars  (n = 15) | TRUSST Curriculum   1. Practice using VR transvaginal simulators 2. Communication skills training | - | The simulation-based training curriculum resulted in improved confidence and ability in TVUS scanning, especially regarding a systematic approach and documentation. | Prospective study | 2021 |
| Calhoun et Hume^58^ | Integrated Obstetric Curriculum for Obstetrics and Gynecology Residency, Radiology Residency and Maternal± Fetal Medicine Fellowshipprogram at an Accredited American Institute of Ultrasound in Medicine Diagnostic Ultrasound Center | OB/GYN | Residents  (n = 13) | Multifaceted training program   1. Structured reading program 2. Self study of a 35-mm slide program of normal/ abnormal anatomy 3. A basic ULS and fetal echocardiography interactive CD program   hands-on supervised scanning program   1. Practical and certificate-bearing fetal echocardiography courses for fellows | - | An integrated approach to Ob ULS training for obstetric and radiologic residents and maternal-fetal medicine fellows with multifaceted learning methods is easily achieved. | Prospective study | 2000 |
| Celebi et al.^59^ | Student Tutors Are Able to Teach Basic Sonographic Anatomy Effectively – a Prospective Randomized Controlled Trial | US education | Medical students  (n = 50) | 1. Group, teached by student tutors | 1. Group, teached by faculty staff | Basic abdominal sonographic anatomy can be taught effectively by student tutors | Prospective randomized study | 2012 |
| Chalouhi et al.^60^ | Evaluation of trainees’ ability to perform obstetrical ultrasound using simulation: challenges and opportunities | OB/GYN | Trainees  (n = 29) | To test the validity of an OB ULS simulator as a tool for evaluating trainees   1. US planes, trainees obtained on an OB ULS simulator | 1. US planes, trainees obtained from volunteers | An OB ULS simulator is as effective as volunteer-based examination for evaluating practical skills of trainees following structured training in OB US. | Prospective observational study | 2015 |
| Dornhofer et al.^61^ | Evaluation of a point-of-care ultrasound curriculum taught by medical students for physicians, nurses, and midwives in rural Indonesia | POCUS | Health-care practitioners  (n = 55) | 4-week POCUS course teached by medical students, followed by postinstructional testing | - | medical students are able to effectively teach POCUS to physicians, nurses, and midwives | Prospective observational study | 2020 |
| Cook et al.^62^ | Simulation-based clinical learning for the third year medical student: Effectiveness of transabdominal and transvaginal ultrasound for elucidation of OB/GYN scenarios | OB/GYN | Medical students  (n = 68) | Simulation-based training with the instructor using both the transabdominal and transvaginal probes | - | This study suggests that ULS simulators are useful for improvement in knowledge, comfort level, and ability to identify pathology in OB/GYN scenarios in third-year medical students. | Prospective Study | 2020 |
| Dromey et al.^63^ | Dimensionless squared jerk: An objective differential to assess experienced and novice probe movement in obstetric ultrasound | OB/GYN | 10 experienced and 10 novice sonographers  (n = 20) | Recorded probe motion data during the performance of biometry on a commercially available mid-trimester phantom   1. experts (n = 10) | 1. Novices (n = 10) | Experienced operator performance was associated with a shorter time to task completion. Probe travel was also shorter for experienced operators when compared to novice operators. | Prospective observational study | 2021 |
| Dyre et al.^64^ | Imperfect practice makes perfect: error management training improves transfer of learning | OB/GYN | Medical students with no prior ULS experience  (n = 60) | Error management training (EMT)  (n = 32) | Error avoidance training (EAT)  (n = 28) | The provision of error management instructions during simulation-based training improves the transfer of learning to the clinical setting compared with error avoidance instructions. | Prospective randomized study | 2017 |
| Dyre et al.^65^ | Collecting Validity Evidence for the Assessment of Mastery Learning in Simulation-Based Ultrasound Training | OB/GYN | 20 novices, 9 ULS experts  (n = 29) | 1. Novices (n = 20) completed 10 OB training modules on a transabdominal ULS simulator that provided simulator metrics | 2. Experts (n = 9) | This study found validity evidence for the assessment of mastery learning in simulation-based ULS training and that ULS novices can attain mastery learning levels with less than 5 hours of training. Only one-third of the standard simulator metrics discriminated between different levels of competence. | Cross-sectional study | 2016 |
| Enabudoso and Adams^66^ | Organizing an international-standard obstetric ultrasonography training program in a low-resource setting | OB/GYN | Participants  (n = 64) | 2-week OB ULS training | - | Several aspects of the workshop and improvements in participant knowledge were rated as good. | Prospective study | 2017 |
| Etienne et al.^67^ | Benefits of using a simulator in the initial training for transvaginal ultrasound examination in gynecologic emergency unit. | OB/GYN | Medical students  (n = 56) | 1. Simulation session in addition to the companionship-training (n = 29) | 1. Only trained by companionship (n = 27) | Using a simulator for medical student was beneficial in the initial training TVUS examination. | Prospective randomized study | 2021 |
| Fung et al.^68^ | An Internet-based learning portfolio in resident education: the KOALA multicenter program | OB/GYN | Residents  (n = 41) | School 1: KOALA prototype for 1 year (a multicenter, Internet-based learning portfolio)  (n1 = 15) | Schools 2, 3, and 4: KOALA-naive schools  (n2,3,4 = 26) | This Internet-based, multi-user, multicenter learning portfolio has a significant effect on residents' perception of their self-directed learning abilities. | Prospective study | 2000 |
| Garcia-Gasasola et al.^69^ | Basic Abdominal Point-of-Care Ultrasound Training in the Undergraduate | Abdominal US | Medical students  (n = 136) | 30 student mentors had to teach fourth-year students (n = 136) the same training they had received | - | Peer teaching achieved an adequate level of training in basic abdominal ULS and the students acquired these skills in a relatively short training period. These results suggest that peer mentoring can facilitate the large-scale implementation of ULS teaching in undergraduate students. | Prospective observational study | 2016 |
| Gardner et al.^70^ | Obstetric Simulation as a Risk Control Strategy Course Design and Evaluation | OB/GYN | 250 OBs, 75 certified nurse midwives, 100 OB anesthesiologists, and 500 L&D nurses  (n = 925) | Systematic design of course development, implementation, and evaluation in 3 phases, including a 1-year or more post training follow-up with self-assessment questionnaires | - | A simulation-based team-training course for obstetric clinicians was developed and is a central component of CRICO/RMF’s obstetric risk management incentive program that provides a 10% reduction in annual obstetrical malpractice premiums. The course was highly regarded immediately and 1 year or more after completing the course. Most survey responders reported improved teamwork and communication in managing a critical obstetric event in the interval since taking the course. Simulation-based CRM training can serve as a strategy for mitigating adverse perinatal events. | Prospective study | 2008 |
| Graber et al.^71^ | Does Simulator Training for Medical Students Change Patient Opinions and Attitudes toward Medical Student Procedures in the Emergency Department? | Emergency medicine (EM) | Patients in the EM department  (n = 151) | Survey | - | Patients are more accepting of medical students performing procedures if the skill has been mastered on a simulator. However, many patients do not want a medical student to perform a procedure on them regardless of the student’s level of training. | Prospective study | 2005 |
| Granados and Wulf^72^ | Enhancing Motor Learning Through Dyad Practice | Research for Exercise and Sport | / | Practice of speed cup stacking under one of four conditions:   1. observation/dialogue 2. observation/no dialogue 3. no observation/dialogue 4. no observation/no dialogue | - | The two conditions that included observational practice were more effective (i.e., produced faster movement times) than the two conditions without it, both during practice and on a retention, test performed under individual performance conditions. | Prospective study | 2007 |
| Grandjean et al.^73^ | Fetal biometry in ultrasound: a new approach to assess the long-term impact of simulation on learning patterns | OB/GYN | Medical students  (n = 61) | 6-month course in OB ULS with SBE-workshop  (simulation-based education)  (n = 39) | 6-month course in OB ULS without SBE-workshop  (n = 22) | The quantitative assessment does not support the existence of long-term benefits from SBE training, although the qualitative assessment confirmed SBE helped to raise the minimal level within a group when embedded in an 'early' stage of a practical course. | Prospective randomized study | 2021 |
| Green et al.^74^ | Obstetric and Gynecologic Resident Ultrasound Education Project | OB/GYN | Residents and physicians  (n = 315) | Survey | - | Current OB/GYN ULS education varies across Canadian residency programs, Training in Gyn ULS is lacking, highlighting the need for a standardized Gyn ULS curriculum for residency programs | Cross-sectional study | 2015 |
| Hall et al.^75^ | Analysis of an obstetrics point-of-care ultrasound training program for healthcare practitioners in Zanzibar, Tanzania | OB/GYN | physicians, clinical officers, and nurse/midwives  (n = 13) | 1. 2-week OB ULS course (lectures and hands-on practice) 2. 6 months of direct supervision of hands-on scanning and bedside education in their clinical environment | - | Trainees improved significantly on all measures after the training program. The low completion rate reflects the challenges of establishing ULS capacity in this type of setting. Further study is needed to determine trainees’ long-term retention of ultrasound skills and the impact of the program on clinical practice and health outcomes. | Prospective observational study | 2021 |
| Hamza et al.^76^ | Introduction of basic obstetrical ultrasound screening in undergraduate medical education | OB/GYN | Medical students  (n = 109) | Additional theoretical and practical course involving hands-on ULS screening during their mandatory practical training week in OB/GYN | - | Using practical, hands-on medical teaching is an emerging method for undergraduate education that should be further evaluated, standardized, and developed. | Prospective study | 2016 |
| Hamza et al.^77^ | Introduction of a student tutor‑based basic obstetrical ultrasound screening in undergraduate medical education | OB/GYN | Medical students  (n = 111) | After a 3 week of training the student tutors, they joined an undergraduate ULS educational program to teach practical round student | - | Significant improvement in theoretical knowledge and fetal image recognition skills  students were satisfied with the course, and indicated that they wanted more ULS hands-on training in both OB/GYN and other medical fields | Prospective study | 2019 |
| Hani et al.^78^ | Introduction of Ultrasound Simulation in Medical Education: Exploratory Study | Multispecialty | Physicians, nurses, ULS technicians, residents, and medical students  (n = 41) | A day-long interactive ULS simulation workshop | - | There was a positive perception toward the use of simulation for training and teaching medical students and residents, and there was a definite need and enthusiasm for its integration into curricula. | Prospective study | 2019 |
| Heer et al.^79^ | Ultrasound training: the virtual patient | OB/GYN | 1. US-experienced Gyns (n = 25) 2. Unexperienced medical students (n = 24) | 1. Experienced Gyns examined 3 virtual cases 🡪 to test the congruence of a live gyn ULS examination and the VR ULS examination 2. Medical students trained with the VR patient | - | VR similar to performing live GYN ULS investigation and allows standardized ULS teaching and learning.  Measurements by all students deviated minimally from the actual measurement. | Prospective study | 2004 |
| Holmlund et al.^80^ | Health professionals’ experiences and views on obstetric ultrasound in Rwanda: A cross-sectional study | OB/GYN | OBs, other physicians, midwives and nurses  (n = 907) | Survey | - | Variable access to ULS depending on health facility level and insufficient skills of ULS operators 🡪 Physicians in general need more training to perform ULS examinations | Cross-sectional study | 2018 |
| Holmlund et al.^81^ | Health professionals’ experiences and views on obstetric ultrasound in Vietnam: a regional, crosssectional study | OB/GYN | OB/GYNs and midwives  (n = 824) | Survey | - | Reports of insufficient ULS training resulting in suboptimal pregnancy management indicate a need for additional training of ULS operators | Cross-sectional study | 2019 |
| Katz et al.^82^ | Simulation Training: Evaluating the Instructor’s Contribution to a Wizard of Oz Simulator in Obstetrics and Gynecology Ultrasound Training | OB/GYN | 1. Group: OB/GYN fellows (n = 18) 2. Group: Senior ULS technicians (n = 14)   (n = 32) | WOZ training session   1. Group n = 8 2. Group n = 7 | E-learning session   1. Group n = 10 2. Group n = 7 | A significant advantage was found in favor of the WOZ training approach.  Involvement of an instructor in the simulation-based training process provided better learning outcomes that varied training content and trainee populations did not affect the overall learning gains. | Prospective randomized study | 2017 |
| Kessler and Bhandarkar^83^ | Ultrasound Training for Medical Students and Internal Medicine Residents—A Needs Assessment | Internal Medicine /  US education | Medical students and residents  (n = 203) | Survey | - | a clear desire for ULS training in among the medical students and internal medicine residents  students and residents would strongly benefit from formal didactic and hands-on training in the modality | Cross-sectional study | 2010 |
| Kim et al.^84^ | The clinical practice patterns of fetal ultrasonography in the first-trimester: A questionnaire survey of members of the Korean Society of Ultrasound in Obstetrics and Gynecology | OB/GYN | Korean Society of Ultrasound in Obstetrics and Gynecology members  (n = 194) | Survey | - | Highlights the need for the practical recommendation or educational course for first-trimester US | Cross-sectional study | 2014 |
| Kim et al.^85^ | An evaluation of obstetric ultrasound education program in Nepal using the REAIM framework | OB/GYN | Physicians and non-physician healthcare workers  (n = 228) | OB ULS education program, evaluated using the REAIM framework | - | The program improved participant’s knowledge and self-confidence in ultrasound techniques and  and showed great potential for the adoption and maintenance of the techniques in their practice | Prospective mixed-method study | 2021 |
| Knobe et al.^86^ | Peer teaching: a randomised controlled trial using student-teachers to teach musculoskeletal ultrasound | Musculoskeletal US | Medical students  (n = 151) | 1. Peer-assisted learning group (PG) using student teachers (STs) | 1. staff-led-group (SG) | US skills can be adequately taught to students by STs with limited training.  But STs still face prejudice from students regarding competency | Prospective randomized study | 2010 |
| Kodikara et al.^87^ | Evaluation of Pacific obstetric and gynaecological ultrasound scanning capabilities, personnel, equipment and workloads | OB/GYN | Sonographers, OB/GYNs, Radiologists, Physicians  (n = 30) | Survey | - | A multimodal training program, incorporating a practical hands-on course, combined with CD/ published materials appears to be the best method of developing more advanced skills in order to optimize antenatal care in the region. | Cross-sectional study | 2010 |
| Lee et al.^88^ | Interactive mulitmedia for prenatal ultrasound training | OB/GYN  Radiology | Physicians  (n = 35) | Survey | - | - Positive feedback for perceived instructional value, question content, subjects covered, graphics interface, and ease of use - A desire for more image-based questions through this multimedia interface - Usefulness of the heart model for spatial orientation, and the broad exposure to various ultrasound anomalies compared to traditional methods | Cross-sectional study | 1995 |
| Lee et al.^89^ | Fetal Ultrasound Training for Obstetrics and Gynecology Residents | OB/GYN | US program directors  (n = 136) | Survey | - | Most significant educational activities: Hands-on scanning and observation   - Learning obstacles: limited curriculum and faculty time - Competency assessment: by direct observation of scanning skills | Cross-sectional study | 2004 |
| Le Lous et al.^90^ | Improving the quality of transvaginal ultrasound scan by simulation training for general practice residents | General practice | General practice residents  (n = 163) | 1. One-day simulation-based ULS training session (n = 137) | 1. No training session (n = 26) | Simulation-based training improved the quality of pelvic ULS images in GP residents assessed after 2 months of experience in gynecology compared to clinical training alone. | Prospective interventional study | 2017 |
| Le Lous et al.^91^ | Impact of Physician Expertise on Probe Trajectory During Obstetric Ultrasound: A Quantitative Approach for Skill Assessment | OB/GYN | 5 experts, 12 intermediates, 16 novices  (n = 33) | Evaluation of the different experienced groups in performing 3 tasks to identify discriminating metrics |  | Our results suggest a relationship between the sonographer's level of expertise and probe trajectory metrics 🡪 Simulator metrics as an indicator/automatic analysis of sonographer proficiency | Prospective observational study | 2021 |
| Madsen et al.^92^ | Assessment of performance measures and learning curves for use of a virtual-reality ultrasound simulator in transvaginal ultrasound examination | OB/GYN | US novices and OB/GYN consultants  (n = 28) | US novices  (n = 16) | OB/GYN consultants  (n = 12) | Competence in the performance of GYN ULS examination can be assessed in a valid and reliable way using VR simulation. The novices’ performance improved with practice and their learning curves plateaued at the level of expert performance, following between 3 and 4 h of simulator training. | Experimental study | 2014 |
| Madsen et al.^93^ | The Predictive Value of Ultrasound Learning Curves Across Simulated and Clinical Settings | OB/GYN | Midwives  (n = 20) | 3-step-training   1. VR simulator training TVUS (n = 20) 2. Mannequin training TVUS (n = 20) 3. Clinical training TVUS (n = 6) | - | Performances during simulation-based sonography training may predict performance in related tasks and subsequent clinical learning curves | Associational study | 2017 |
| Mattar and Gribble^94^ | Motor Learning by Observing | Neuroscience | Subjects  (n = 24) | 1. Observation group (n = 12) | 1. No observation (n = 12) | Mechanisms matching observation and action facilitate motor learning. | Experimental study | 2005 |
| Maul et al.^95^ | Ultrasound simulators: experience with the SonoTrainer and comparative review of other training systems | OB/GYN | Certified OBs  (n = 45) | 1. Theoretical training and ULS simulator (n = 21) | 1. Only theoretical training (n = 24) | Simulator-based training could provide an ideal educational tool to test, improve and monitor a physician’s or technician’s ULS skills in detecting fetal anomalies. | Prospective study and review | 2004 |
| McCurdy et al.^96^ | Transvaginal ultrasound training for the obstetrics and gynecology resident: A multisite randomized controlled trial of educational pocket brain | OB/GYN | Residents  (n = 57) | 1. Educational DVD | 1. Routine education | An educational DVD is easily implemented and demonstrates short-term benefit. | Prospective randomized study | 2018 |
| Moak et al.^97^ | Training in Transvaginal Sonography Using Pelvic Ultrasound Simulators Versus Live Models: A Randomized Controlled Trial | OB/GYN | Medical students  (n = 134) | 1. TVUS training using an ULS simulator (n = 62) | 1. TVUS using live models (n = 72) | Simulators do not perform as well as LMs for training novices in TVS, but they may be useful as an adjunct to LM training. | Prospective randomized study | 2014 |
| Mukamel et al.^98^ | Single neuron responses in humans during execution and observation of actions | Neuroscience | Patients  (n =21) | Single neuron responses in humans during execution and observation of actions were recorded | - | These findings suggest that multiple systems in humans may be endowed with neural mechanisms of mirroring for both the integration and differentiation of perceptual and motor aspects of actions performed by self and others. | Experimental study | 2010 |
| Nicholls et al.^99^ | A survey of Australian sonographer psychomotor teaching practices | - | Sonographers  (n = 528) | Survey | - | - A pressing need to identify the optimal pedagogical approaches to teach complex psychomotor scanning skills - Research is required to teach these skills effectively - an accompanying need for sonographers involved in teaching scanning skills to have knowledge of the motor-learning theories and principles related to teaching a complex psychomotor skill | Cross-sectional study | 2020 |
| Noerholk et al.^100^ | Does group size matter during collaborative skills learning? A randomised study | OB/GYN | Medical students  (n = 101) | 1. Single training (n = 24) (2h of OB ULS training) | 1. Dyad training (n = 26) 2. Triad training (n = 27) 3. Tetrad training (n = 24) | Group size did not significantly influence performance measured on the transfer test. | Prospective randomized study | 2022 |
| Ooi et al.^101^ | Reaudit of transvaginal ultrasound practice in a general gynecology clinic | GYN | GYN ULS examinations  (n = 48) | Reaudit: Observations of completed GYN ULS practice | initial audit, which found, that the quality of practice in a teaching hospital did not reflect current guidelines | Regular audit, ad hoc ULS training sessions and updated ULS equipment resulted in considerably improved compliance of transvaginal ultrasound practice in gynecology. | Prospective observational study | 2020 |
| Patel et al.^102^ | The Role of Ultrasound Simulation in Obstetrics and Gynecology Training: A UK Trainees’ Perspective | OB/GYN | OB/GYN trainees  (n = 70) | Survey | - | Trainees are struggling to achieve minimal ULS competences with clinical ULS training alone. They believe that ULS simulation will shorten the learning curve and improve their clinical skills and knowledge. | Cross-sectional study | 2016 |
| Popowski et al.^103^ | Impact of Accreditation Training for Residents on Sonographic Quality in Gynecologic Emergencies | OB/GYN | OB/GYN residents  (n = 10) | 1. Residents with accreditation training (n = 5) | 2. Residents without accreditation training  (n = 5) | An accreditation training process including facilitated feedback from a local opinion leader improved the quality of ULS examinations performed | Prospective study | 2015 |
| Recker et al.^104^ | Development and implementation of a comprehensive postgraduate ultrasound curriculum for residents in obstetrics and gynecology: a feasibility study | OB/GYN | 1st and 2nd year assistant physicians  (n = 14) | Theoretical and practical ULS basic course (six modules) in addition to their obligatory clinic rotations  (n =14) | - | The course is feasible and improved the learning curve of young OB/GYN residents rapidly. | Prospective study | 2022 |
| Rosen et al.^105^ | Simulator Based Obstetric Ultrasound Training: A Prospective, Randomized Single-Blinded Study. | OB/GYN | OB trainees  (n = 18) | 1. Simulator group (n = 9) | 1. Patient group (n = 9) | Simulation-based training performed as well as real patient training and was found to be especially beneficial for beginner trainees.  SBME has a high rate of acceptance by trainees, does not require investment of patient or clinic resources, and warrants consideration as an educational tool for the safe and effective teaching of OB US. | Prospective randomized study | 2017 |
| Sanchez-Ku et al.^106^ | A Dyadic Protocol for Training Complex Skills: A Replication Using Female Participants | Psychology / Medical education | Female undergraduate students  (n = 108) | 1. AlM-dyad condition (active interlocked modeling dyadic protocol) (n = 54) | 1. Standard individual control training condition (n = 54) | Women trained in the AIM-dyad condition performed as well as those trained in the individual condition. Thus, the efficiency gains associated with the AIM dyad protocol, which result from the ability to train two people simultaneously to reach the same performance level as a single person with no increase in training time or machine cost, are generalizable to female participants. | Prospective randomized study | 2000 |
| Shaw-Battista et al.^107^ | Interprofessional Obstetric Ultrasound Education: Successful Development of Online Learning Modules; Case-Based Seminars; and Skills Labs for Registered and Advanced Practice Nurses, Midwives, Physicians, and Trainees | OB/GYN | Interprofessional (Advanced Practice Nurses, Midwives, Physicians, and Trainees)  (n = 162) | OB ULS course, including seminars, skills lab training and asynchronous e-learning modules | - | Course evaluations were extremely positive: online modules were an excellent means to develop basic knowledge; the seminars and particularly the hands-on skills labs were also key to their learning | Prospective study | 2015 |
| Shea et al.^108^ | Enhancing Training Efficiency and Effectiveness Through the Use of Dyad Training | Psychology / Medical education | Students  (n = 36) | 1. Individual practice (n=12) | 1. Dyad-alternate practice (n=12) 2. Dyad-control practice (n=12) | The results suggest that one can combine the benefits of physical practice, observation, and dialog between learners in an interactive way to produce an effective and efficient learning protocol. | Prospective randomized study | 1999 |
| Staboulidou et al.^109^ | Quality assured ultrasound simulator training for the detection of fetal malformations | OB/GYN | Experienced OBs and Gyns  (n = 1266) | Survey  after the advanced Simulator based ULS training course to detect the most common malformations | - | Simulation-based ULS training courses seem to be useful for defining a basic standardized quality of training and significantly improving examiners’ skills. This is a suitable additional instrument to improve the education in OB US. | Prospective study | 2010 |
| Tolsgaard et al.^110^ | Improving Efficiency of Clinical Skills Training: A Randomized Trial | - | Medical students  (n = 49) | 1. Dyad practice group (n = 24) | 1. Single practice group (n=25) | Dyad training of pre-clerkship medical students’ patient encounter skills is effective, efficient, and prompts higher confidence in managing future patient encounters compared to training alone. | Prospective randomized study | 2013 |
| Tolsgaard et al.^111^ | International Multispecialty Consensus on How to Evaluate Ultrasound Competence: A Delphi Consensus Survey | Multispecialty | US experts  (n = 60) | 3-round Delphi questionnaire |  | International multispecialty consensus was achieved on the content of a generic ultrasound rating scale (OSAUS) | Cross-sectional study | 2013 |
| Tolsgaard et al.^112^ | Feasibility of self-directed learning in clerkships | Medical education | Medical students  (n = 498) | To explore the feasibility of self-directed learning stimulated by clinical encounter-cards (CECs) in clinical clerkships  🡪 students were interviewed about the usefulness of CECs to their learning in clerkships |  | Students rated the usefulness of the CECs on learning in clerkship low along with preceptor support | Prospective study | 2013 |
| Tolsgaard et al.^113^ | Medical students’ perception of dyad practice | - | Medical students  (n = 24) | follow-up pilot survey to 24 fourth-year medical students, who completed four hours of dyad practice in managing patient encounters | - | Dyad practice is well received by students during initial skills training and is associated with several benefits to learning through peer observation, feedback and cognitive support. | Exploratory qualitative research study | 2014 |
| Tolsgaard et al.^114^ | Reliable and valid assessment of ultrasound operator competence in obstetrics and gynecology | OB/GYN | Physicians with different levels of ULS experience in OB/GYN  (n = 30) | 1. Novice (n = 10)   🡪15 performed transabdominal OB scans and 15 TVUS GYN scans | 1. Intermediate group (n = 10) 2. Senior group (n = 10) | US competence can be assessed in a reliable and valid way using the OSAUS scale. The pass/fail scores may be used to help determine when trainees are qualified for independent practice. | Cross-sectional study | 2014 |
| Tolsgaard et al.^115^ | Which factors are associated with trainees‘ confidence in performing obstetric and gynecological ultrasound examinations? | OB/GYN | Trainees  (n = 621) | Survey | - | Clinical experience and  time spent in specialized ultrasound units were predictors  of trainees’ confidence in performing ULS independently.  Three factors were related to ULS confidence: technical aspects,  image perception and integration of scan into patient care.  Discrepancies between trainees’ confidence and their expected levels of performance raised concerns about the adequacy of current ultrasound training programs. | Cross-sectional study | 2014 |
| Tolsgaard et al.^116^ | Linking quality of care and training costs: cost-effectiveness in health professions education | OB/GYN | Midwives  (n = 12) | CLM (cervical length measurement) training 🡪 midwife performed CLMs  (n = 6) | No training 🡪 initial management by midwife, and CLM performed by OB  (n = 6) | Women who were scanned by interventiongroup participants had significantly reduced waiting time compared with those managed by the control group.  Different training strategies could be recommended as the most cost-effective depending on administrators’ willingness to pay per unit of the outcome variable. | Prospective randomized study and Cost-Effectiveness Analysis | 2015 |
| Tolsgaard et al.^117^ | The effect of dyad versus individual simulation-based ultrasound training on skills transfer | OB/GYN | Medical students  (n = 30) | 1. Dyad practice group (n = 16) | 1. Single practice group (n = 14) | Dyad practice improves the efficiency of simulation-based training and is non-inferior to individual practice in terms of skills transfer | Prospective randomized study | 2015 |
| Tolsgaard et al.^118^ | Sustained effect of simulation-based ultrasound training  on clinical performance: a randomized trial | OB/GYN | Residents  (n = 33) | Simulation-based training and subsequent clinical training  (n = 18) | Clinical training only  (n = 15) | Simulation-based ultrasound training leads to substantial improvement in clinical performance that is sustained after 2 months of clinical training. | Prospective randomized study | 2015 |
| Tolsgaard et al.^119^ | The Effects of Simulation-based Transvaginal Ultrasound Training on Quality and Efficiency of Care | OB/GYN | Trainees  (n = 54) | Simulation-based ultrasound training and clinical training  (n = 28) | Clinical training only  (n = 26) | Simulation-based ultrasound training improved quality of care and reduced the need for repeated patient examination and trainee supervision. | Prospective randomized study | 2017 |
| Tregonning et al.^120^ | The audience response system and knowledge gain: a prospective study | OB/GYN | Medical students  (n = 170) | Audience response system (ARS) format | Didactic lecture | Use of the ARS in lectures appeared to improve knowledge gain immediately post-lecture but no difference was found after retesting at 5 weeks. | Prospective study | 2012 |
| Vrachnis et al.^121^ | International Society of Ultrasound in Obstetrics and Gynecology (ISUOG) - the propagation of knowledge in ultrasound for the improvement of OB/GYN care worldwide: experience of basic ultrasound training in Oman | OB/GYN | Sonolographers from OB/GYN, fetal medicine and general medicine with different experience levels  (n = 28) | 5 day course  (each day 4h theoretcial lectures and 4h practical training) | - | Improved the theoretical knowledge and practical skills of local health personnel (only short-term impact measured) | Prospective study | 2019 |
| Vyas et al.^122^ | Feasibility study of minimally trained medical students using the Rural Obstetrical Ultrasound Triage Exam (ROUTE) in rural Panama | OB/GYN | First-year medical students  (n=8) | First-year medical students completed ROUTE training sessions and then performed the ROUTE in mobile clinics  (n=8) |  | Based on our data, first-year medical students with additional training can use the ROUTE to identify complications in pregnancy using ultrasound in rural Panama. | Prospective observational study | 2018 |
| Windrim and Higgins^123^ | Trans-vaginal ultrasound simulation: An exploratory qualitative research study focused on the end-users perception of learning | OB/GYN | Physicians with no previous experience in TVUS  (n = 15) | 3h training session on the transvaginal simulator and a teaching program  1. individual training (n=7) | 2. dyad training  (n = 4 pairs, eight people in total) | The participants appreciated the value of simulation and provided feedback for improvement in educational content.  Clinicians and could consider dyad training as an efficient and educational option, with individual training reserved for those who may need more time. | Prospective study | 2022 |
| Yang et al.^124^ | Learning curve in measurement of fetal frontomaxillary facial angle at 11–13 weeks of gestation | OB/GYN | Sonographers  (n = 8) | Training to measure the fetal FMF (frontomaxillary facial) angle using specially acquired 3D-volumes | - | Competence in FMF angle measurement was achieved after a median number of 90 cases, with a range of up to 140 | Prospective study | 2010 |
| Yaqub et al.^125^ | Quality-improvement program for ultrasound-based fetal anatomy screening using large-scale clinical audit | OB/GYN | Sonographers  (n = 20) | 1. Large-scale, clinical, retrospective audit ULS images was conducted 2. Targeted actions in response to the findings 3. A second audit was then performed of fetal anatomy ULS images obtained during the following year |  | A clinical audit and a set of targeted actions helped improve sonographer scan-acquisition completeness and scan quality. | Retrospective study | 2019 |
| Yerra et al.^126^ | Simulation‑Based Training on Basic Obstetrics and Gynecology ULS Skills During COVID Pandemic | OB/GYN | Health care professionals  (n = 80) | 8-day-long workshop | - | Improvement in knowledge, instrument handling, basic GYN skills, and practical ULS skills 🡪 US-based simulation can provide a realistic setting for training and assessment of novices in learning basic skills. | Prospective study | 2023 |

References

1. Abuhamad A, Minton KK, Benson CB, Chudleigh T, Crites L, Doubilet PM, et al. Obstetric and Gynecologic Ultrasound Curriculum and Competency Assessment in Residency Training Programs: Consensus Report. J Ultrasound Med. 2018 Jan;37(1):19–50.

2. Benacerraf BR, Minton KK, Benson CB, Bromley BS, Coley BD, Doubilet PM, et al. Proceedings: Beyond Ultrasound First Forum on Improving the Quality of Ultrasound Imaging in Obstetrics and Gynecology. J Ultrasound Med. 2018 Jan;37(1):7–18.

3. Bidner A, Bezak E, Parange N. Evaluation of antenatal Point-of-Care Ultrasound (PoCUS) training: a systematic review. Med Educ Online. 2022;27(1):2041366.

4. Chalouhi GE, Bernardi V, Ville Y. Ultrasound simulators in obstetrics and gynecology: state of the art. Ultrasound in Obstetrics & Gynecology. 2015;46(3):255–63.

5. Cook DA, Hatala R, Brydges R, Zendejas B, Szostek JH, Wang AT, et al. Technology-enhanced simulation for health professions education: a systematic review and meta-analysis. JAMA. 2011 Sep 7;306(9):978–88.

6. Dietrich CF, Hoffmann B, Abramowicz J, Badea R, Braden B, Cantisani V, et al. Medical Student Ultrasound Education: A WFUMB Position Paper, Part I. Ultrasound in Medicine & Biology. 2019 Feb;45(2):271–81.

7. Dromey BP, Peebles DM, Stoyanov DV. A Systematic Review and Meta-analysis of the Use of High-Fidelity Simulation in Obstetric Ultrasound. Simulation in Healthcare. 2021 Feb;16(1):52.

8. Ericsson KA, Krampe RT, Tesch-Römer C. The role of deliberate practice in the acquisition of expert performance. Psychological Review. 19931101;100(3):363.

9. Gaba DM. The future vision of simulation in health care. Quality and Safety in Health Care. 2004 Oct 1;13(suppl_1):i2–10.

10. Gabor P, Dimassi K, Aabakke AJM, Rouveau R, Ami O. Ultrasound training in obstetrics and gynecology in Europe: satisfaction survey. Ultrasound in Obstetrics & Gynecology. 2018;51(4):559–60.

11. Giacomino K, Caliesch R, Sattelmayer KM. The effectiveness of the Peyton’s 4-step teaching approach on skill acquisition of procedures in health professions education: A systematic review and meta-analysis with integrated meta-regression. PeerJ. 2020 Oct 9;8:e10129.

12. Hillerup NE, Tabor A, Konge L, Savran MM, Tolsgaard MG. Validity of ISUOG basic training test. Ultrasound in Obstetrics & Gynecology. 2018;52(2):279–80.

13. Hoffmann B, Blaivas M, Abramowicz J, Bachmann M, Badea R, Braden B, et al. Medical Student Ultrasound Education, a WFUMB Position Paper, Part II. A consensus statement of ultrasound societies. Med Ultrason. 2020 May 11;22(2):220.

14. Hoppmann RA, Rao VV, Poston MB, Howe DB, Hunt PS, Fowler SD, et al. An integrated ultrasound curriculum (iUSC) for medical students: 4-year experience. Crit Ultrasound J. 2011 Apr;3(1):1–12.

15. Höhne E, Recker F, Dietrich CF, Schäfer VS. Assessment Methods in Medical Ultrasound Education. Front Med (Lausanne). 2022 Jun 9;9:871957.

16. Issenberg SB, McGaghie WC, Petrusa ER, Gordon DL, Scalese RJ. Features and uses of high-fidelity medical simulations that lead to effective learning: a BEME systematic review: Medical Teacher: Vol 27, No 1 [Internet]. 2005 [cited 2023 Apr 21]. Available from: https://www.tandfonline.com/doi/abs/10.1080/01421590500046924

17. ISUOG Education Committee recommendations for basic training in obstetric and gynecological ultrasound. Ultrasound in Obstetrics & Gynecology. 2014;43(1):113–6.

18. Lammers S, Dolin CD, Baston C. A Call for Development of Point-of-Care Ultrasound Training Recommendations in Obstetrics and Gynecology Residency. Journal of Ultrasound in Medicine. 2022;41(7):1845–8.

19. Lous ML, Klein M, Tesson C, Berthelemy J, Lavoue V, Jannin P. Metrics used to evaluate obstetric ultrasound skills on simulators: A systematic review. European Journal of Obstetrics and Gynecology and Reproductive Biology. 2021 Mar 1;258:16–22.

20. Leonardi M, Murji A, D’Souza R. Ultrasound curricula in obstetrics and gynecology training programs. Ultrasound in Obstetrics & Gynecology. 2018;52(2):147–50.

21. McGaghie WC, Issenberg SB, Petrusa ER, Scalese RJ. A critical review of simulation-based medical education research: 2003–2009. Medical Education. 2010;44(1):50–63.

22. Nicholls D, Sweet L, Hyett J. Psychomotor Skills in Medical Ultrasound Imaging. Journal of Ultrasound in Medicine. 2014;33(8):1349–52.

23. Nicholls D, Sweet L, Muller A, Hyett J. Teaching psychomotor skills in the twenty-first century: Revisiting and reviewing instructional approaches through the lens of contemporary literature. Medical Teacher. 2016 Oct 2;38(10):1056–63.

24. Nielsen M, Cantisani V, Sidhu P, Badea R, Batko T, Carlsen J, et al. The Use of Handheld Ultrasound Devices – An EFSUMB Position Paper. Ultraschall in Med. 2019 Feb;40(01):30–9.

25. Nitsche JF, Brost BC. Obstetric ultrasound simulation. Semin Perinatol. 2013 Jun;37(3):199–204.

26. Okuda Y, Bryson EO, DeMaria Jr S, Jacobson L, Quinones J, Shen B, et al. The Utility of Simulation in Medical Education: What Is the Evidence? Mount Sinai Journal of Medicine: A Journal of Translational and Personalized Medicine. 2009;76(4):330–43.

27. Recker F, Weber E, Strizek B, Gembruch U, Westerway SC, Dietrich CF. Point-of-care ultrasound in obstetrics and gynecology. Arch Gynecol Obstet. 2021;303(4):871–6.

28. Rizzolatti G, Craighero L. The mirror-neuron system. Annu Rev Neurosci. 2004;27:169–92.

29. Salvesen KA, Lees C, Tutschek B. Basic European ultrasound training in obstetrics and gynecology: where are we and where do we go from here? Ultrasound Obstet Gynecol. 2010 Nov;36(5):525–9.

30. Scalese RJ, Obeso VT, Issenberg SB. Simulation Technology for Skills Training and Competency Assessment in Medical Education. J GEN INTERN MED. 2008 Jan;23(S1):46–9.

31. Stone J, Abu-Rustum RS, Bromley B, Fuchs KM, Anton T, Cooper T, et al. Curriculum and Competency Assessment Program for Training Maternal-Fetal Medicine Fellows in the Performance of the Detailed Obstetric Ultrasound Examination. Journal of Ultrasound in Medicine. 2022;41(12):2925–32.

32. Sweller J. Cognitive Load During Problem Solving: Effects on Learning. Cognitive Science. 1988;12(2):257–85.

33. Taksøe-Vester C, Dyre L, Schroll J, Tabor A, Tolsgaard M. Simulation-Based Ultrasound Training in Obstetrics and Gynecology: A Systematic Review and Meta-Analysis. Ultraschall Med. 2021 Dec;42(6):e42–54.

34. Tolsgaard MG, Kulasegaram KM, Ringsted CV. Collaborative learning of clinical skills in health professions education: the why, how, when and for whom. Medical Education. 2016;50(1):69–78.

35. Tolsgaard MG, Chalouhi GE. Use of ultrasound simulators for assessment of trainee competence: trendy toys or valuable instruments? Ultrasound in Obstetrics & Gynecology. 2018;52(4):424–6.

36. Tolsgaard MG. Assessment and learning of ultrasound skills in Obstetrics & Gynecology. Dan Med J. 2018 Feb;65(2):B5445.

37. Topping KJ. Trends in Peer Learning. Educational Psychology. 2005 Dec 1;25(6):631–45.

38. Tsai PJS, Wong S, Zalud I, Izutsu S. Ultrasound Education in Obstetrics and Gynecology: Hawai‘i Experience. Hawaii J Med Public Health. 2013 May;72(5):172–4.

39. Tutschek B, Pilu G. Pocket Brain, an interactive, web-based ultrasound atlas of normal and abnormal fetal brain development. Ultrasound in Obstetrics & Gynecology. 2017;49(4):431–2.

40. Van der Aa J, Goverde A, Scheele F. Improving the training of the future gynaecologist: development of a European curriculum in Obstetrics and Gynaecology (EBCOG-PACT). Facts Views Vis Obgyn. 10(1):1–2.

41. Viner AC, Okolo ID, Norman JE, Stock SJ, Reynolds RM. Training in Ultrasound to Determine Gestational Age in Low- and Middle- Income Countries: A Systematic Review. Front Glob Womens Health. 2022 Mar 18;3:854198.

42. Vrachnis N, Cohen-Overbeek TE, Collaborators. ISUOG ultrasound training in Oman: evaluating participant long-term retention and effect of repetitive practical courses on ultrasound knowledge and skills. Ultrasound in Obstetrics & Gynecology. 2021;58(2):175–7.

43. Woodhead NJ, Mahmud A, Clark J. Effects of simulation for gynaecological ultrasound scan training: a systematic review. BMJ Simul Technol Enhanc Learn. 2020 Nov 1;6(6):320–31.

44. Wulf G, Shea C, Lewthwaite R. Motor skill learning and performance: a review of influential factors. Medical Education. 2010;44(1):75–84.

45. Zaidi S. Obstetrics and Gynaecology in the Asia and Oceania Region: Requirements for Postgraduate Training and Certification. Journal of Obstetrics and Gynaecology Research. 1999;25(2):71–85.

46. Ziv A, Wolpe PR, Small SD, Glick S. Simulation-Based Medical Education: An Ethical Imperative. Simulation in Healthcare: The Journal of the Society for Simulation in Healthcare. 2003;1(4):252–6.

47. Ahmadzia H, Cigna S, Namagembe I, Macri C, Galerneau F, Magriples U. Teaching obstetric ultrasound at Mulago Hospital - Kampala, Uganda. Afr Health Sci. 2018 Mar;18(1):166–71.

48. Akoma UN, Shumard KM, Street L, Brost BC, Nitsche JF. Impact of an Inexpensive Anatomy-Based Fetal Pig Simulator on Obstetric Ultrasound Training. Journal of Ultrasound in Medicine. 2015;34(10):1793–9.

49. Alrahmani L, Codsi E, Borowski KS. The Current State of Ultrasound Training in Obstetrics and Gynecology Residency Programs. Journal of Ultrasound in Medicine. 2018;37(9):2201–7.

50. Amesse LS, Callendar E, Pfaff-Amesse T, Duke J, Herbert WNP. Evaluation of Computer-aided Strategies for Teaching Medical Students Prenatal Ultrasound Diagnostic Skills. Med Educ Online. 2008 Sep 24;13:13.

51. Andreasen LA, Tabor A, Nørgaard LN, Ringsted C, Sandager P, Rosthøj S, et al. Multicenter randomized trial exploring effects of simulation-based ultrasound training on obstetricians’ diagnostic accuracy: value for experienced operators. Ultrasound in Obstetrics & Gynecology. 2020;55(4):523–9.

52. Arthur Jr. W, Day EA, Bennett Jr. W, McNelly TL, Jordan JA. Dyadic versus individual training protocols: Loss and reacquisition of a complex skill. Journal of Applied Psychology. 19970101;82(5):783.

53. Bahner DP, Royall NA. Advanced Ultrasound Training for Fourth-Year Medical Students: A Novel Training Program at The Ohio State University College of Medicine. Academic Medicine. 2013 Feb;88(2):206–13.

54. Bentley S, Mudan G, Strother C, Wong N. Are Live Ultrasound Models Replaceable? Traditional versus Simulated Education Module for FAST Exam. West J Emerg Med. 2015 Nov;16(6):818–22.

55. Burden C, Preshaw J, White P, Draycott TJ, Grant S, Fox R. Validation of Virtual Reality Simulation for Obstetric Ultrasonography: A Prospective Cross-sectional Study. Simulation in Healthcare: The Journal of the Society for Simulation in Healthcare. 2012 Oct;7(5):269–73.

56. Burden C, Preshaw J, White P, Draycott TJ, Grant S, Fox R. Usability of virtual-reality simulation training in obstetric ultrasonography: a prospective cohort study. Ultrasound in Obstetrics & Gynecology. 2013;42(2):213–7.

57. Byford S, Janssens S, Cook R. Implementing the transvaginal ultrasound simulation training (TRUSST) programme for obstetric registrars. Advances in Simulation. 2021 Jan 12;6(1):1.

58. Calhoun BC, Hume JR RF. Integrated obstetric curriculum for obstetrics and gynecology residency, radiology residency and maternal–fetal medicine fellowship program at an Accredited American Institute of Ultrasound in Medicine Diagnostic Ultrasound Center. Ultrasound in Obstetrics & Gynecology. 2000;16(1):68–71.

59. Celebi N, Zwirner K, Lischner U, Bauder M, Ditthard K, Schürger S, et al. Student Tutors Are Able to Teach Basic Sonographic Anatomy Effectively – a Prospective Randomized Controlled Trial. Ultraschall Med. 2010 Nov 23;141–5.

60. Chalouhi GE, Bernardi V, Gueneuc A, Houssin I, Stirnemann JJ, Ville Y. Evaluation of trainees’ ability to perform obstetrical ultrasound using simulation: challenges and opportunities. American Journal of Obstetrics & Gynecology. 2015 Nov 4;214(4):525.e1-525.e8.

61. Dornhofer K, Farhat A, Guan K, Parker E, Kong C, Kim D, et al. Evaluation of a point-of-care ultrasound curriculum taught by medical students for physicians, nurses, and midwives in rural Indonesia. Journal of Clinical Ultrasound. 2020;48(3):145–51.

62. Cook J, Rao VV, Bell F, Durkin M, Cone J, Lane-Cordova A, et al. Simulation-based clinical learning for the third year medical student: Effectiveness of transabdominal and transvaginal ultrasound for elucidation of OB/GYN scenarios. Journal of Clinical Ultrasound. 2020;48(8):457–61.

63. Dromey BP, Ahmed S, Vasconcelos F, Mazomenos E, Kunpalin Y, Ourselin S, et al. Dimensionless squared jerk: An objective differential to assess experienced and novice probe movement in obstetric ultrasound. Prenatal Diagnosis. 2021;41(2):271–7.

64. Dyre L, Tabor A, Ringsted C, Tolsgaard MG. Imperfect practice makes perfect: error management training improves transfer of learning. Medical Education. 2017;51(2):196–206.

65. Dyre L, Nørgaard LN, Tabor A, Madsen ME, Sørensen JL, Ringsted C, et al. Collecting Validity Evidence for the Assessment of Mastery Learning in Simulation-Based Ultrasound Training. Ultraschall Med. 2016 Aug;37(4):386–92.

66. Enabudoso E, Adams OH. Organizing an international-standard obstetric ultrasonography training program in a low-resource setting. International Journal of Gynecology & Obstetrics. 2017;136(1):102–4.

67. Etienne M, Gabay L, Levaillant JM, Vivanti A, Dommergues M, Fernandez H, et al. Benefits of using a simulator in the initial training for transvaginal ultrasound examination in gynecologic emergency unit. Journal of Gynecology Obstetrics and Human Reproduction. 2021 Feb;50(2):101938.

68. Fung MFK, Walker M, Fung KFK, Temple L, Lajoie F, Bellemare G, et al. An Internet-based learning portfolio in resident education: the KOALA^TM^ multicentre programme. Medical Education. 2000;34(6):474–9.

69. Garcia-Casasola G, Sánchez FJG, Luordo D, Zapata DF, Frías MC, Garrido VV, et al. Basic Abdominal Point-of-Care Ultrasound Training in the Undergraduate. Journal of Ultrasound in Medicine. 2016;35(11):2483–9.

70. Gardner R, Walzer TB, Simon R, Raemer DB. Obstetric simulation as a risk control strategy: course design and evaluation. Simul Healthc. 2008;3(2):119–27.

71. Graber MA, Wyatt C, Kasparek L, Xu Y. Does Simulator Training for Medical Students Change Patient Opinions and Attitudes toward Medical Student Procedures in the Emergency Department? Academic Emergency Medicine. 2005;12(7):635–9.

72. Granados C, Wulf G. Enhancing Motor Learning Through Dyad Practice. Research Quarterly for Exercise and Sport [Internet]. 2007 [cited 2023 Apr 14]; Available from: https://www.tandfonline.com/doi/abs/10.1080/02701367.2007.10599417

73. Grandjean GA, Bertholdt C, Zuily S, Fauvel M, Hossu G, Berveiller P, et al. Fetal biometry in ultrasound: A new approach to assess the long-term impact of simulation on learning patterns. Journal of Gynecology Obstetrics and Human Reproduction. 2021 Oct;50(8):102135.

74. Green J, Kahan M, Wong S. Obstetric and Gynecologic Resident Ultrasound Education Project. Journal of Ultrasound in Medicine. 2015;34(9):1583–9.

75. Hall EA, Matilsky D, Zang R, Hase N, Habibu Ali A, Henwood PC, et al. Analysis of an obstetrics point-of-care ultrasound training program for healthcare practitioners in Zanzibar, Tanzania. Ultrasound J. 2021 Apr 8;13:18.

76. Hamza A, Solomayer EF, Takacs Z, Juhasz-Boes I, Joukhadar R, Radosa JC, et al. Introduction of basic obstetrical ultrasound screening in undergraduate medical education. Arch Gynecol Obstet. 2016 Sep;294(3):479–85.

77. Hamza A, Radosa JC, Solomayer EF, Takacs Z, Juhasz-Boess I, Ströder R, et al. Introduction of a student tutor-based basic obstetrical ultrasound screening in undergraduate medical education. Arch Gynecol Obstet. 2019 Jul;300(1):59–66.

78. Hani S, Chalouhi G, Lakissian Z, Sharara-Chami R. Introduction of Ultrasound Simulation in Medical Education: Exploratory Study. JMIR Med Educ. 2019 Sep 26;5(2):e13568.

79. Heer IM, Middendorf K, Müller-Egloff S, Dugas M, Strauss A. Ultrasound training: the virtual patient. Ultrasound in Obstetrics & Gynecology. 2004;24(4):440–4.

80. Holmlund S, Ntaganira J, Edvardsson K, Lan PT, Semasaka Sengoma JP, Lesio Kidanto H, et al. Health professionals’ experiences and views on obstetric ultrasound in Rwanda: A cross-sectional study. PLoS One. 2018 Dec 4;13(12):e0208387.

81. Holmlund S, Lan PT, Edvardsson K, Phuc HD, Ntaganira J, Small R, et al. Health professionals’ experiences and views on obstetric ultrasound in Vietnam: a regional, cross-sectional study. BMJ Open. 2019 Sep;9(9):e031761.

82. Katz A, Tepper R, Shtub A. Simulation Training: Evaluating the Instructor’s Contribution to a Wizard of Oz Simulator in Obstetrics and Gynecology Ultrasound Training. JMIR Med Educ. 2017 Apr 21;3(1):e8.

83. Kessler C, Bhandarkar S. Ultrasound training for medical students and internal medicine residents—A needs assessment. Journal of Clinical Ultrasound. 2010;38(8):401–8.

84. Kim KW, Kwak DW, Ko HS, Park HS, Seol HJ, Hong JS, et al. The clinical practice patterns of fetal ultrasonography in the first-trimester: A questionnaire survey of members of the Korean Society of Ultrasound in Obstetrics and Gynecology. Obstet Gynecol Sci. 2014 Nov;57(6):448–56.

85. Kim J, Shakya PR, Choi S, Park JS, Tamrakar SR, Heo J, et al. An evaluation of obstetric ultrasound education program in Nepal using the RE-AIM framework. BMC Medical Education. 2021 Jan 15;21(1):57.

86. Knobe M, Münker R, Sellei RM, Holschen M, Mooij SC, Schmidt-Rohlfing B, et al. Peer teaching: a randomised controlled trial using student-teachers to teach musculoskeletal ultrasound. Medical Education. 2010;44(2):148–55.

87. Kodikara H, Mitchell J, Ekeroma A, Stone P. Evaluation of Pacific obstetric and gynaecological ultrasound scanning capabilities, personnel, equipment and workloads. The New Zealand medical journal. 2010 Dec 17;123:58–67.

88. Lee W. Interactive multimedia for prenatal ultrasound training. Obstetrics & Gynecology. 1995 Jan;85(1):135–41.

89. Lee W, Hodges AN, Williams S, Vettraino IM, McNie B. Fetal Ultrasound Training for Obstetrics and Gynecology Residents. Obstetrics & Gynecology. 2004 Feb;103(2):333.

90. Le Lous M, De Chanaud N, Bourret A, Senat MV, Colmant C, Jaury P, et al. Improving the quality of transvaginal ultrasound scan by simulation training for general practice residents. Advances in Simulation. 2017 Nov 21;2(1):24.

91. Le Lous M, Despinoy F, Klein M, Fustec E, Lavoue V, Jannin P. Impact of Physician Expertise on Probe Trajectory During Obstetric Ultrasound: A Quantitative Approach for Skill Assessment. Sim Healthcare. 2021 Feb;16(1):67–72.

92. Madsen ME, Konge L, Nørgaard LN, Tabor A, Ringsted C, Klemmensen ÅK, et al. Assessment of performance measures and learning curves for use of a virtual-reality ultrasound simulator in transvaginal ultrasound examination. Ultrasound in Obstetrics & Gynecology. 2014;44(6):693–9.

93. Madsen ME, Nørgaard LN, Tabor A, Konge L, Ringsted C, Tolsgaard MG. The Predictive Value of Ultrasound Learning Curves Across Simulated and Clinical Settings. Journal of Ultrasound in Medicine. 2017;36(1):201–8.

94. Mattar AAG, Gribble PL. Motor Learning by Observing. Neuron. 2005 Apr 7;46(1):153–60.

95. Maul H, Scharf A, Baier P, Wüstemann M, Günter HH, Gebauer G, et al. Ultrasound simulators: experience with the SonoTrainer and comparative review of other training systems. Ultrasound in Obstetrics & Gynecology. 2004;24(5):581–5.

96. McCurdy RJ, High B, Schnatz PF, Baxter J, Jiang X. Transvaginal ultrasound training for the obstetrics and gynecology resident: A multisite randomized controlled trial of educational DVD. Journal of Clinical Ultrasound. 2018;47(2):71–6.

97. Moak JH, Larese SR, Riordan JP, Sudhir A, Yan G. Training in Transvaginal Sonography Using Pelvic Ultrasound Simulators Versus Live Models: A Randomized Controlled Trial. Academic Medicine. 2014 Jul;89(7):1063.

98. Mukamel R, Ekstrom AD, Kaplan J, Iacoboni M, Fried I. Single neuron responses in humans during execution and observation of actions. Curr Biol. 2010 Apr 27;20(8):750–6.

99. Nicholls D, Sweet L, Hyett J, Müller A. A survey of Australian sonographer psychomotor teaching practices. Australas J Ultrasound Med. 2020 Aug 18;23(4):227–37.

100. Noerholk LM, Morcke AM, Kulasegaram K, Nørgaard LN, Harmsen L, Andreasen LA, et al. Does group size matter during collaborative skills learning? A randomised study. Medical Education. 2022;56(6):680–9.

101. Ooi R, Ooi S, Wilson D, Griffiths A. Reaudit of transvaginal ultrasound practice in a general gynecology clinic. Journal of Clinical Ultrasound. 2020;48(6):312–4.

102. Patel H, Chandrasekaran D, Myriokefalitaki E, Gebeh A, Jones K, Jeve YB. The Role of Ultrasound Simulation in Obstetrics and Gynecology Training: A UK Trainees’ Perspective. Sim Healthcare. 2016 Oct;11(5):340–4.

103. Popowski T, Huchon C, Fathallah K, Falissard B, Dumont A, Fauconnier A. Impact of Accreditation Training for Residents on Sonographic Quality in Gynecologic Emergencies. Journal of Ultrasound in Medicine. 2015;34(5):829–35.

104. Recker F, Dugar M, Böckenhoff P, Gembruch U, Geipel A. Development and implementation of a comprehensive postgraduate ultrasound curriculum for residents in obstetrics and gynecology: a feasibility study. Arch Gynecol Obstet. 2022 Oct;306(4):1045–51.

105. Rosen H, Windrim R, Lee YM, Gotha L, Perelman V, Ronzoni S. Simulator Based Obstetric Ultrasound Training: A Prospective, Randomized Single-Blinded Study. Journal of Obstetrics and Gynaecology Canada. 2017 Mar 1;39(3):166–73.

106. Sanchez-Ku ML. A Dyadic Protocol for Training Complex Skills: A Replication Using Female Participants [Internet]. 2000 [cited 2023 Apr 14]. Available from: https://journals.sagepub.com/doi/epdf/10.1518/001872000779698169

107. Shaw-Battista J, Young-Lin N, Bearman S, Dau K, Vargas J. Interprofessional Obstetric Ultrasound Education: Successful Development of Online Learning Modules; Case-Based Seminars; and Skills Labs for Registered and Advanced Practice Nurses, Midwives, Physicians, and Trainees. Journal of Midwifery & Women’s Health. 2015;60(6):727–34.

108. Shea CH, Wulf G, Whltacre C. Enhancing Training Efficiency and Effectiveness Through the Use of Dyad Training. Journal of Motor Behavior [Internet]. 1999 [cited 2023 Apr 14]; Available from: https://www.tandfonline.com/doi/abs/10.1080/00222899909600983

109. Staboulidou I, Wüstemann M, Vaske B, Elsässer M, Hillemanns P, Scharf A. Quality assured ultrasound simulator training for the detection of fetal malformations. Acta Obstetricia et Gynecologica Scandinavica. 2010;89(3):350–4.

110. Tolsgaard MG, Bjørck S, Rasmussen MB, Gustafsson A, Ringsted C. Improving Efficiency of Clinical Skills Training: A Randomized Trial. J Gen Intern Med. 2013 Aug;28(8):1072–7.

111. Tolsgaard MG, Todsen T, Sorensen JL, Ringsted C, Lorentzen T, Ottesen B, et al. International Multispecialty Consensus on How to Evaluate Ultrasound Competence: A Delphi Consensus Survey. PLoS One. 2013 Feb 28;8(2):e57687.

112. Tolsgaard MG, Arendrup H, Pedersen P, Ringsted C. Feasibility of self-directed learning in clerkships. Medical Teacher. 2013 Aug 1;35(8):e1409–15.

113. Tolsgaard MG, Rasmussen MB, Bjørck S, Gustafsson A, Ringsted CV. Medical students’ perception of dyad practice. Perspect Med Educ. 2014 Dec;3(6):500–7.

114. Tolsgaard MG, Ringsted C, Dreisler E, Klemmensen A, Loft A, Sorensen JL, et al. Reliable and valid assessment of ultrasound operator competence in obstetrics and gynecology. Ultrasound in Obstetrics & Gynecology. 2014;43(4):437–43.

115. Tolsgaard MG, Rasmussen MB, Tappert C, Sundler M, Sorensen JL, Ottesen B, et al. Which factors are associated with trainees’ confidence in performing obstetric and gynecological ultrasound examinations? Ultrasound in Obstetrics & Gynecology. 2014;43(4):444–51.

116. Tolsgaard MG, Tabor A, Madsen ME, Wulff CB, Dyre L, Ringsted C, et al. Linking quality of care and training costs: cost-effectiveness in health professions education. Medical Education. 2015;49(12):1263–71.

117. Tolsgaard MG, Madsen ME, Ringsted C, Oxlund BS, Oldenburg A, Sorensen JL, et al. The effect of dyad versus individual simulation-based ultrasound training on skills transfer. Medical Education. 2015;49(3):286–95.

118. Tolsgaard MG, Ringsted C, Dreisler E, Nørgaard LN, Petersen JH, Madsen ME, et al. Sustained effect of simulation-based ultrasound training on clinical performance: a randomized trial. Ultrasound in Obstetrics & Gynecology. 2015;46(3):312–8.

119. Tolsgaard MG, Ringsted C, Rosthøj S, Nørgaard L, Møller L, Freiesleben NLC, et al. The Effects of Simulation-based Transvaginal Ultrasound Training on Quality and Efficiency of Care: A Multicenter Single-blind Randomized Trial. Annals of Surgery. 2017 Mar;265(3):630.

120. Tregonning AM, Doherty DA, Hornbuckle J, Dickinson JE. The audience response system and knowledge gain: A prospective study. 2012 [cited 2023 May 20]; Available from: https://www.tandfonline.com/doi/epdf/10.3109/0142159X.2012.660218?needAccess=true&role=button

121. Vrachnis N, Papageorghiou AT, Bilardo CM, Abuhamad A, Tabor A, Cohen-Overbeek TE, et al. International Society of Ultrasound in Obstetrics and Gynecology (ISUOG) - the propagation of knowledge in ultrasound for the improvement of OB/GYN care worldwide: experience of basic ultrasound training in Oman. BMC Med Educ. 2019 Dec;19(1):434.

122. Vyas A, Moran K, Livingston J, Gonzales S, Torres M, Duffens A, et al. Feasibility study of minimally trained medical students using the Rural Obstetrical Ultrasound Triage Exam (ROUTE) in rural Panama. World J Emerg Med. 2018;9(3):216–22.

123. Windrim C, Higgins MF. Trans-vaginal ultrasound simulation: An exploratory qualitative research study focused on the end-users perception of learning. European Journal of Obstetrics & Gynecology and Reproductive Biology. 2022 Mar;270:201–5.

124. Yang X, Chen M, Wang HF, Leung TY, Borenstein M, Nicolaides K, et al. Learning curve in measurement of fetal frontomaxillary facial angle at 11–13 weeks of gestation. Ultrasound in Obstetrics & Gynecology. 2010;35(5):530–4.

125. Yaqub M, Kelly B, Stobart H, Napolitano R, Noble JA, Papageorghiou AT. Quality-improvement program for ultrasound-based fetal anatomy screening using large-scale clinical audit. Ultrasound in Obstetrics & Gynecology. 2019;54(2):239–45.

126. Yerra AK, Jogi S, Emmadisetty S, Animalla V, D’souza A. Simulation-Based Training on Basic Obstetrics and Gynecology Ultrasound Skills During COVID Pandemic. J Obstet Gynaecol India. 2023 Jan 7;1–6.
